# The Brain Tumor Segmentation (BraTS-METS) Challenge 2023: Brain Metastasis Segmentation on Pre-treatment MRI

**Published:** 2023-06-01

**Authors:** Ahmed W. Moawad, Anastasia Janas, Ujjwal Baid, Divya Ramakrishnan, Leon Jekel, Kiril Krantchev, Harrison Moy, Rachit Saluja, Klara Osenberg, Klara Wilms, Manpreet Kaur, Arman Avesta, Gabriel Cassinelli Pedersen, Nazanin Maleki, Mahdi Salimi, Sarah Merkaj, Marc von Reppert, Niklas Tillmans, Jan Lost, Khaled Bousabarah, Wolfgang Holler, MingDe Lin, Malte Westerhoff, Ryan Maresca, Katherine E. Link, Nourel hoda Tahon, Daniel Marcus, Aristeidis Sotiras, Pamela LaMontagne, Strajit Chakrabarty, Oleg Teytelboym, Ayda Youssef, Ayaman Nada, Yuri S. Velichko, Nicolo Gennaro, Connectome Students, Group of Annotators, Justin Cramer, Derek R. Johnson, Benjamin Y.M. Kwan, Boyan Petrovic, Satya N. Patro, Lei Wu, Tiffany So, Gerry Thompson, Anthony Kam, Gloria Guzman Perez-Carrillo, Neil Lall, Group of Approvers, Jake Albrecht, Udunna Anazodo, Marius George Lingaru, Bjoern H Menze, Benedikt Wiestler, Maruf Adewole, Syed Muhammad Anwar, Dominic Labella, Hongwei Bran Li, Juan Eugenio Iglesias, Keyvan Farahani, James Eddy, Timothy Bergquist, Verena Chung, Russel Takeshi Shinohara, Farouk Dako, Walter Wiggins, Zachary Reitman, Chunhao Wang, Xinyang Liu, Zhifan Jiang, Koen Van Leemput, Marie Piraud, Ivan Ezhov, Elaine Johanson, Zeke Meier, Ariana Familiar, Anahita Fathi Kazerooni, Florian Kofler, Evan Calabrese, Sanjay Aneja, Veronica Chiang, Ichiro Ikuta, Umber Shafique, Fatima Memon, Gian Marco Conte, Spyridon Bakas, Jeffrey Rudie, Mariam Aboian

**Affiliations:** 1. Mercy Catholic Medical Center, Darby, PA; 2. Yale University School of Medicine, Department of Radiology, New Haven, CT; 3. ImagineQuant, Yale University School of Medicine, Department of Radiology, New Haven, CT; 4. Charité - Universitatsmedizin, Berlin, Germany; 5. Center for Biomedical Image Computing and Analytics, University of Pennsylvania School of Medicine, Philadelphia, PA; 6. Department of Radiology, Perelman School of Medicine, University of Pennsylvania, Philadelphia, PA; 7. DKFZ Division of Translational Neurooncology at the WTZ, German Cancer Consortium, DKTK Partner Site, University Hospital Essen, Essen, Germany; 8. German Cancer Research Center, Heidelberg, Germany; 9. Cornell University, Ithaca, NY; 10. University of Leipzig, Leipzig, Germany; 11. Ludwig Maximillian University, Munich, Germany; 12. University of Ulm, Ulm, Germany; 13. University of Dusseldorf, Medical Faculty, Department of Diagnostic and Interventional Radiology, Dusseldorf, Germany; 14. Visage Imaging, GmbH, Berlin, Germany; 15. Visage Imaging, Inc, San Diego, California, USA; 16. Yale University School of Medicine, Department of Therapeutic Radiology, New Haven, CT; 18. New York University School of Medicine, New York, NY; 19. University of Missouri, Columbia, MI; 20. Washington University, St. Louis, MI; 21. National Cancer Institute, Cairo, Egypt; 22. Northwestern University, Department of Radiology, Feinberg School of Medicine, Chicago, IL; 23. Connectome – Student Association for Neurosurgery, Neurology and Neurosciences E.V.; 24. Neuroradiologists from ASNR with expertise in neuroimaging; 25. Mayo Clinic, Phoenix, Arizona; 26. Department of Radiology, Mayo Clinic, Rochester, MN; 27. Queen’s University, Department of Diagnostic Radiology, Kingston, Canada; 28. NorthShore University HealthSystem, Evanston, IL.; 29. University of Arkansas for Medical Sciences, Little Rock, AR, USA; 30. University of Washington Department of Radiology, Seattle, WA; 31. Department of Imaging and Interventional Radiology, Faculty of Medicine, The Chinese University of Hong Kong; 32. The University of Edinburgh, Edinburgh, Scotland; 33. Loyola University Medical Center, Chicago, IL; 34. Washington University Department of Radiology, St. Louis, MO; 35. Children’s Healthcare of Atlanta, Atlanta, GA; 36. Sage Bionetworks, USA; 37. Montreal Neurological Institute (MNI), McGill University, Montreal, CA; 38. Children’s National Hospital, Washington DC, USA; 39. Biomedical Image Analysis & Machine Learning, Department of Quantitative Biomedicine, University of Zurich, Switzerland; 40. Department of Neuroradiology, Technical University of Munich, Munich, Germany; 41. Medical Artificial Intelligence (MAI) Lab, Crestview Radiology, Lagos, Nigeria; 42. Duke University School of Medicine, Durham, NC; 43. Athinoula A. Martinos Center for Biomedical Imaging, Massachusetts General Hospital, Boston, MA; 44. Cancer Imaging Program, National Cancer Institute, National Institutes of Health, Bethesda, MD; 45. Center for Clinical Epidemiology and Biostatistics, University of Pennsylvania, Philadelphia, PA; 46. Center for Global Health, Perelman School of Medicine, University of Pennsylvania, Philadelphia, PA; 47. Department of Applied Mathematics and Computer Science, Technical University of Denmark, Denmark; 48. Helmholtz AI, Helmholtz Munich, Germany; 49. Department of Informatics, Technical University Munich, Germany; 50. PrecisionFDA, U.S. Food and Drug Administration, Silver Spring, MD; 51. Booz Allen Hamilton, McLean, VA; 52. Children’s Hospital of Philadelphia, University of Pennsylvania, Philadelphia, PA; 53. Technische Universität München, Munich, Germany; 54. Yale University School of Medicine, Department of Neurosurgery, New Haven, CT; 55. Indiana University School of Medicine, Indianapolis, IN; 56. University of California San Diego, San Diego, CA; 57. University of California San Francisco, San Francisco, CA

**Keywords:** BraTS, BraTS-METS, challenge, brain, tumor, segmentation, machine learning, artificial intelligence, AI

## Abstract

Clinical monitoring of metastatic disease to the brain can be a laborious and timeconsuming process, especially in cases involving multiple metastases when the assessment is performed manually. The Response Assessment in Neuro-Oncology Brain Metastases (RANO-BM) guideline, which utilizes the unidimensional longest diameter, is commonly used in clinical and research settings to evaluate response to therapy in patients with brain metastases. However, accurate volumetric assessment of the lesion and surrounding peri-lesional edema holds significant importance in clinical decision-making and can greatly enhance outcome prediction. The unique challenge in performing segmentations of brain metastases lies in their common occurrence as small lesions. Detection and segmentation of lesions that are smaller than 10 mm in size has not demonstrated high accuracy in prior publications. The brain metastases challenge sets itself apart from previously conducted MICCAI challenges on glioma segmentation due to the significant variability in lesion size. Unlike gliomas, which tend to be larger on presentation scans, brain metastases exhibit a wide range of sizes and tend to include small lesions. We hope that the BraTS-METS dataset and challenge will advance the field of automated brain metastasis detection and segmentation.

## Introduction

Brain metastases are the most common malignancy affecting the central nervous system (CNS) in adults. The evaluation of brain metastases in clinical practice is commonly limited to comparison to only one prior imaging study due to the frequent occurrence of multiple metastases in a single patient. Comparison to multiple prior studies can be done for some of the lesions but is commonly performed in select cases. This usually occurs when there are specific concerns or changes observed in the patient’s condition. For example, the mixed or atypical response to treatment, appearance of the metastasis, or the need for accurate monitoring of the metastatic lesions over time. Therefore, there is a critical need for multi-lesion segmentation and treatment follow-up over multiple studies. However, achieving this goal effectively requires the use of automatic algorithms that detect and segment metastases on multiple imaging time points, including pre- and post-treatment scans.^1,2^ Detailed analysis of multiple patient lesions on multiple serial scans is impossible in current clinical practice because of the time it requires to assess a study and variability in techniques used at different time points. It is common for follow-up imaging to be performed using different scanners and for different radiologists to review images from different time points for the same patient. This introduces additional factors such as acquisition heterogeneity and inter-reader variability. Therefore, the development of automated segmentation tools for brain metastases that is applicable to multiple practice settings and variable techniques is essential for providing a high level of patient care and consistent measurements.

Brain metastases can appear anywhere in the brain and can be of any size. However, brain metastases that are smaller than 10 mm in diameter are more common than large ones. Accurate detection of small metastatic lesions, which are typically 1–2 mm in size, is critical for patient prognosis. Missing even a single lesion can result in patient requiring repeat interventions, experiencing delay in treatment, and incurring increased costs. Gross total volume of brain metastases is an important predictor of patient outcomes, but it is not currently available in clinical practice due to the lack of volumetric segmentation tools and the time required to detect and accurately perform volumetric segmentation of all lesions, regardless of size.^3^ Many of the segmentation algorithms that were developed for gliomas, such as nnU-Net, demonstrate high accuracy assessed using Dice scores for larger metastases. However, their performance^3–13^ for small metastases is lower as compared to larger lesions.^4–13^ Addressing this challenge is critically important for the development of novel segmentation and detection algorithms specifically designed for brain metastases that are common in clinical practice. By successfully overcoming this challenge, we can provide algorithms that can be readily translated and implemented in clinical settings.

**Figure 1:**
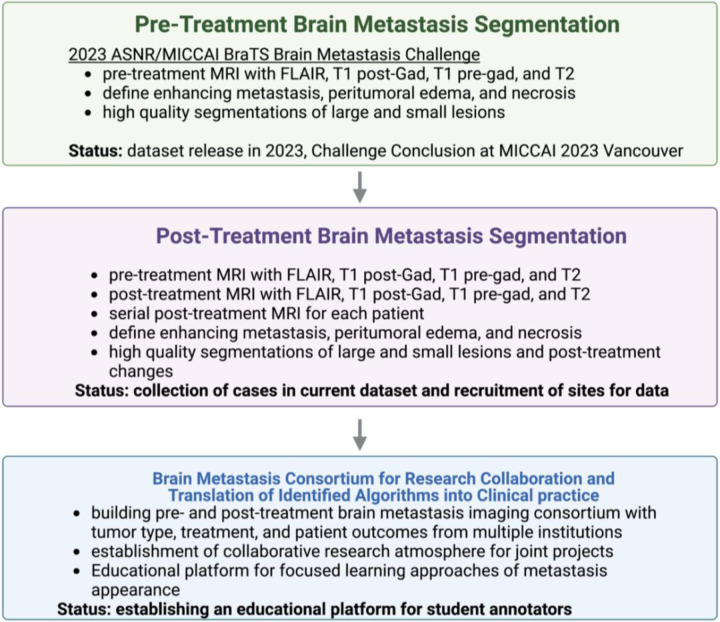
Flow chart for the vision for the BraTS-METS Brain Metastasis Challenge which starts with initial 2023 ASNR MICCAI BraTS Challenge for pre-treatment brain metastases diagnosed on MRI brain. During this challenge high quality segmentations were performed on a small subset of collected datasets to optimize the data set for algorithm development by the MICCAI Challenge participants. The dataset will be expanded in the follow up challenges with continual annotation process of the contributed Brain MRI. Follow up challenges will also start including datasets that contain high quality annotated data of post-treated brain metastases, additional segmentations that include hemorrhagic component of the tumor, and using non-skull stripped images to optimize evaluation of dural-based and osseous metastases. These images will be curated with clinical data and patient demographics and will be included into an inter-institutional consortium of Brain Metastases that encourages collaborative discoveries and translation of algorithms into clinical practice with academic and industry collaborative approach.

We plan to address this problem in two phases (Figure 1). In Phase 1, we will develop an algorithm for segmentation of pre-treated brain metastases. The algorithm will be optimized for detection and segmentation of the enhancing portion, peri-tumoral edema, and necrotic portion of brain metastases. In Phase 2, we will develop a segmentation algorithm for post-treated brain metastases. This algorithm will consider longitudinal changes of individual lesions over the course of multiple studies.

Previous BraTS challenges have been focused only on adult brain diffuse astrocytoma.^14–16^ However, the focus of the BraTS cluster of challenges for the year 2023 has expanded to include various new brain tumor entitites, addressing missing data and technical considerations. Specifically, the challenge described here is focused on brain metastases that are imaged prior to the initiation of treatment. The primary objective of BraTS-METS 2023 Challenge is to facilitate identification of challenge participant-developed solutions for a segmentation algorithm capable of accurately segmenting both large and small metastases on diagnostic Magnetic Resonance Imaging (MRI), utilizing T1 pre-contrast, T1 post-contrast, T2, and FLAIR sequences. This will provide a standardized autosegmentation algorithm that will be open source and available to institutions, enabling its seamless incorporation into their clinical and research workflows.

## Materials & Methods

### Data

The BraTS-METS dataset is a retrospective collection of brain tumor multiparametric MRI (mpMRI) scans acquired from multiple different institutions under standard clinical conditions. However, these scans were acquired with different equipment and imaging protocols, resulting in a vastly heterogeneous image quality reflecting diverse clinical practice across different institutions. Inclusion criteria comprised presence of untreated brain metastases on MRI with T1 pre-contrast, T1 postcontrast, T2, and T2-FLAIR sequences. The data contributors were responsible for submission IRB and DTA to their individual institution regulating bodies and obtaining approval. Upon receiving approval from the IRB and approval of the DTA, data was centralized and curated.

Exclusion criteria include presence of prior treatment changes or no visible brain metastases, lack of one of four MRI sequences, and the presence of significant motion or imaging artifacts. The cases where post-treatment changes are noted are being reserved for the 2024 BraTS-METS Brain Metastasis challenge that will include these cases. The image annotation process is reviewed below.

Following the paradigm of algorithmic evaluation in machine learning, the data included in the BraTS-METS 2023 challenge are divided into training (70%), validation (10%), and testing datasets (20%). The challenge participants are provided with the ground truth labels only for the training data. The validation data are then provided to the participants without any associated ground truth and the testing data are kept hidden from the participants at all times. Participants are not allowed to use additional public and/or private data (from their own institutions) for extending the provided BraTS-METS data, for the training of the algorithm chosen to be ranked. Similarly, using models that were pretrained on such datasets is not allowed. This is due to our intention to provide a fair comparison among the participating methods. However, participants are allowed to use additional public and/or private data (from their own institutions), only for scientific publication purposes and if they explicitly mention this in their submitted manuscripts. Importantly, participants that decide to proceed with this scientific analysis must also report results using only the BraTS-METS 2023 data to discuss potential result differences.

#### Imaging Data Description

The mpMRI scans included in the BraTS-METS 2023 challenge describe a) T1-weighted (T1), b) post-gadolinium contrast T1-weighted (T1Gd), c) T2-weighted (T2), and d) T2 Fluid Attenuated Inversion Recovery (T2-FLAIR) volumes, acquired with different protocols and various scanners from multiple institutions.

Standardized pre-processing has been applied to all the BraTS-METS mpMRI scans. Specifically, the applied pre-processing routines include conversion of the DICOM files to the NIfTI file format, co-registration to the same anatomical template (SRI24)^17^, resampling to a uniform isotropic resolution (1 mm3), and finally skull-stripping. The pre-processing pipeline is publicly available through the Cancer Imaging Phenomics Toolkit (CaPTk)^18,19^ and Federated Tumor Segmentation (FeTS) tool^20,21^. Conversion to NIfTI strips the accompanying metadata from the DICOM images, and essentially removes all Protected Health Information (PHI) from the DICOM headers. Furthermore, skull-stripping mitigates potential facial reconstruction/recognition of the patient^22,23^. The specific approach we have used for skull stripping is based on a novel DL approach that accounts for the brain shape prior and is agnostic to the MRI sequence input.^24,25^

**Figure 2:**
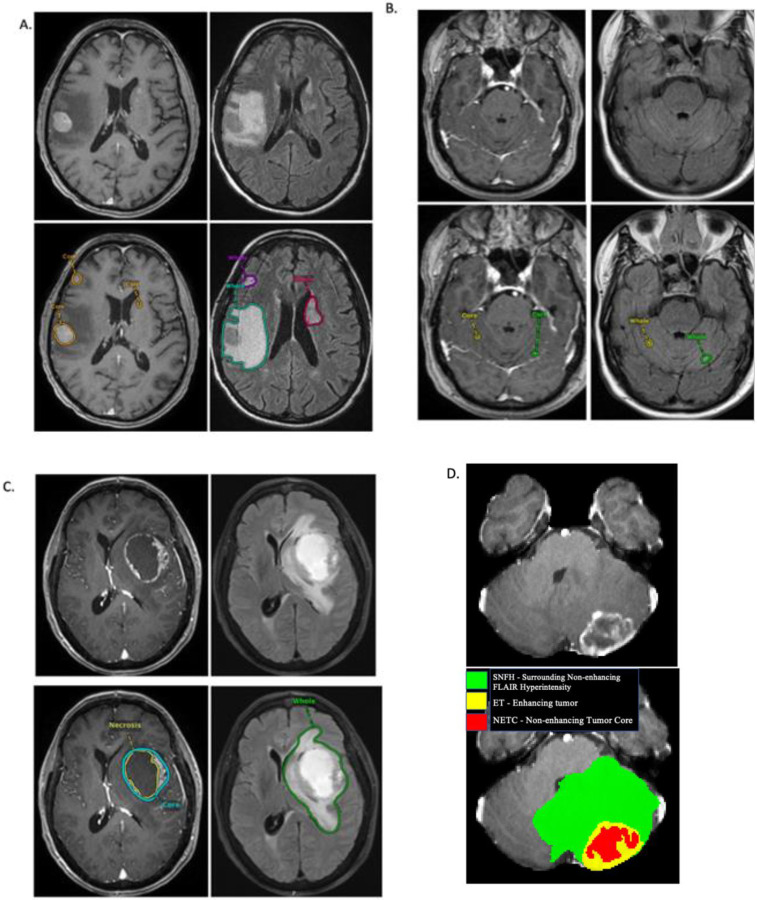
Image panels with the tumor sub-regions annotated in the different mpMRI scans. The image panels A denote the regions considered for the performance evaluation of the participating algorithms and specifically highlight: the enhancing tumor (ET - orange) visible in a T1Gd scan and surrounding edema indicating Surrounding Non-enhancing FLAIR Hyperintensity (SNFH - green, purple, and red). Multiple lesions of various sizes are noted in panel A demonstrating the complexity of annotation of each scan. In panel B, small lesions located within the cerebellar hemispheres are identified and enhancing tumor and surrounding FLAIR hyperintense region are identified on segmentations. In panel C, Visage PACS image of a large centrally necrotic tumor is shown within the left frontal lobe with segmentations of the enhancing tumor (ET), central necrotic portion (SNFH), and peritumoral edema (SNFH). In panel D, ITK-SNAP image of Enhancing tumor (ET) (yellow), Surrounding Non-enhancing FLAIR Hyperintensity (SNFH) (green), and Non-enhancing Tumor Core (NETC) (red) segmentations are demonstrated.

All imaging volumes have then been segmented using a) nnU-Net trained on University of California, San Francisco Brain Metastases Stereotactic Radiosurgery (UCSF-BMSR) MRI Dataset^26,27^, b) U-Net trained on AURORA multicenter study^7^, c) nnU-Net trained on Heidelberg University Hospital data set.^28^ The three segmentations have been fused using combination of STAPLE fusion algorithm and voting based algorithm for each label.^29^ All segmentations, as well as fused segmentations, were provided to the annotators. Subtraction images, where T1-weighted sequence is digitally subtracted from the post contrast sequence, are provided as well to the annotators to facilitate annotation process. These labels were refined manually by volunteer neuroradiology experts of varying rank and experience, following a consistently communicated annotation protocol. The manually refined annotations were finally approved by experienced board-certified attending neuro-radiologists, with more than 15 years of experience working with brain tumors. The annotated tumor sub-regions are based upon known observations visible to the trained radiologist (VASARI features) and comprise the Gd-enhancing tumor (Enhancing tumor (ET) - label 3), the peritumoral edematous/infiltrated tissue (Surrounding non-enhancing FLAIR hyperintensity (SNFH) - label 2), and the nonenhancing tumor core (NETC – label 1). ET is the enhancing portion of the tumor, described by areas with both visually avid, as well as faint, enhancement on T1Gd MRI. NETC is the presumed necrotic core of the tumor, the appearance of which is hypointense on T1Gd MRI with surrounding contrast enhancement. SNFH is the peritumoral edema and tumor infiltrated tissue, defined by the abnormal hyperintense signal on the T2-FLAIR volumes, which includes the infiltrative non enhancing tumor, as well as vasogenic edema in the peritumoral region. In the previous BraTS challenges, ET was segmented as label 4. However, starting from BraTS 2023, ET will be segmented as label 3 for consistency. The sub-regions are shown in Figure 2.

#### Tumor Annotation Protocol:

The BraTS initiative, in consultation with internationally recognized expert neuroradiologists, defined various tumor sub-regions in an attempt to offer a standardized approach to assess and evaluate them. However, other criteria for delineation could be set, resulting in slightly different tumor sub-regions. We designed the following tumor annotation protocol, to ensure consistency in the ground truth delineations across various annotators. Structural mpMRI volumes were considered (T1, T1Gd, T2, T2-FLAIR), all of them co-registered to a common anatomical template (SRI24^17^) and resampled to 1mm^3^. The end-to-end pipeline is available for these through CaPTk^18,19,30^ and FeTS tools^20^.

The BraTS-METS 2023 challenge focuses on three regions of interest:

- Whole Tumor (WT) = Label 1 + Label 2 + Label 3
- Tumor Core (TC) = Label 1 + Label 3
- Enhancing Tumor (ET) = Label 3

The ET is defined as areas of hyperintensity in T1Gd that are brighter than T1 and healthy white matter in T1Gd. The TC is defined as the bulk of the metastasis, which is typically treated with radiotherapy, medical therapy, or surgery. The ET includes the non-enhancing tumor core (NETC), which typically appear hypointense in T1Gd compared to T1. The WT describes the complete extent of the disease, as it includes the ET and the peritumoral edematous/invaded tissue, which is typically depicted by the abnormal hyper-intense signal in the T2-FLAIR volume. However, radiologic definition of tumor boundaries, especially in infiltrative tumors such as gliomas, is a well-known problem. This is less of a problem in brain metastases, which typically have well defined borders of the contrast enhancing portion. In most cases, the boundaries of the contrast enhancing region of the brain metastasis and the surrounding FLAIR hyperintense edema are well defined. The major difficulty in segmenting brain metastases is the overlap of edema between multiple lesions. This is why we separate the segmentation of ET from WT and treat them as separate entities. ET segmentations are not linked to WT segmentations.

To facilitate the annotation process for BraTS-METS 2023, initial automated segmentations were generated using previously developed algorithms.^7,26–28^ The label fusion process was different for each label. The SNFH (label – 2) was fused using the STAPLE fusion algorithm^29^ to aggregate the segmentations produced by each of the individual automated segmentation algorithm and account for systematic errors generated by each of them separately. The ET (label – 3) was fused by the minority voting algorithm to aggregate all enhancing tumor voxels produced by all automated segmentation. This was done because the accuracy of detecting small metastases varied across the automated segmentation algorithms. The NCR (label – 1) is only produced by nnU-Net trained on UCSF-BMSR. Algorithms trained on AURORA and Heidelberg datasets only segment TC and SNFH. Therefore, NCR overlays both ET and SNFH labels.

#### Image Annotation Process:

Pre-segmented images are annotated by student annotators, volunteer neuroradiology experts of varying rank and experience, and reviewed by Annotator Coordinators (A.J. and K.K.). The cases where annotations are incomplete are returned back to the students to re-annotate. During the process of annotation, the volunteer annotators undergo group reviews of cases where they can ask questions and attend course lectures by expert imagers in the field. Once student annotations are complete, they are sent to pool of experienced board-certified attending neuro-radiologists (Group of Approvers, list of individual approvers will be provided in subsequent manuscripts) that were recruited by the ASNR (American Society of Neuroradiology). The approvers review the volunteer annotations and decide to either approve the case or send back to students for annotation. a quality control (QC) process including a) removing all random voxels and any voxels outside the brain mask, b) making sure all images have the same parameters (space, orientation and origin) as SRI24 atlas, and c) making sure all segmentations are present and segmentation masks are in the folder with original NifTI images.

#### Common errors of automated segmentations:

Based on observations from previous BraTS challenges, we have identified some common errors in automated segmentations. The most typical errors in the current challenge are:

- The segmentation of post-treatment lesions. The cases were removed where lesions after surgery or Gamma Knife were segmented.
- The segmentation of white matter changes from microvascular disease. Peritumoral edema segmentations were checked by neuroradiology attendings and modified.
- The segmentation of non-enhancing lesions that have intrinsic T1 hyperintensity. Voxels with intrinsic T1 hyperintensity were manually removed from ET segmentation.

#### Performance Evaluation:

We will offer participants a baseline approach implemented in the Generally Nuanced Deep Learning Framework (GaNDLF),^31^ a modular open-source framework maintained by the MLCommons organization. GaNDLF offers some network architectures, but also allows users to leverage the functionality of other libraries, such as PILLOW and MONAI. Participants can decide to use GaNDLF to develop their approach or use their own custom source code. Submissions must be packaged in an MLCube container, as instructions in the Synapse platform. MLCube containers are automatically generated by GaNDLF and will be used to evaluate all submissions through the MedPerf platform^32^ on each contributing site’s data.

Performance evaluation will be based on Dice scores for individual segmented lesions: enhancing tumor (ET), tumor core (ET and NETC), and whole tumor which includes peritumoral edema (SNFH and ET and NETC). An additional approach for evaluating performance in the BraTS-METS 2023 challenge is lesion-based detection. Dice scores and Hausdorff distances will be computed for individual lesions. Given that brain metastases are typically small, punctate regions comprising only a few voxels, it is clinically significant to assess segmentation algorithms based on their capacity to accurately detect and delineate both small and large lesions. Teams will be ranked based on their scores (a combination of Dice scores and Hausdorff distances).

Future investigations may include evaluating tumor heterogeneity of radiomic features between different phenotypes of brain metastatic patterns and classification tasks such as predicting the metastatic tumor of origin.

#### Participation Timeline:

The challenge will begin with the release of the training dataset on June 1 2023, which will include imaging data and corresponding ground-truth labels. Participants can then design and train their methods using this training dataset. Following the release of the training data, the validation data will be made available. This will enable participants to assess their methods on unseen data and report their preliminary results in their submitted short MICCAI LNCS papers, along with their cross-validated results on the training data. While the ground truth of the validation data will not be provided to participants, they will have the opportunity to make multiple submissions to the online evaluation platforms. The top-ranked teams in the validation phase will be invited to prepare slides for a brief oral presentation of their method during the BraTS-METS challenge at MICCAI 2023.

Finally, all participants will be evaluated and ranked using the same unseen testing data, which will not be accessible to the participants. They will need to upload their containerized method to the evaluation platforms for evaluation. The final top-ranked teams will be announced at the 2023 MICCAI Annual Meeting. Monetary prizes will be awarded to the top-ranked teams in both tasks of the challenge.

## Results

**Figure 3:**
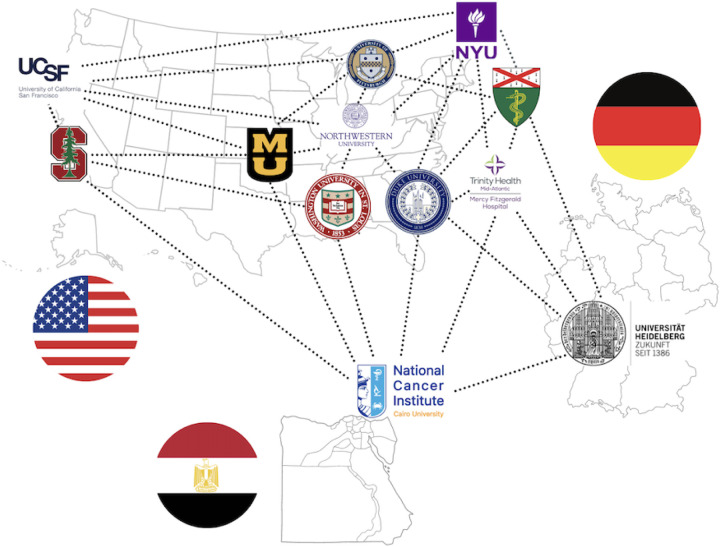
Map of institutions that reached out with interest in contributing data to the ASNR MICCAI BraTS-METS Brain Metastases Challenge. The institutions indicated on the map have submitted IRB and DTA at their individual institutions and at the time of publication are either in the process of being evaluated or are approved.

Multiple datasets were contributed by individual institutions and are in various stages of annotation and approval (Figure 3).

Datasets from NYU, Yale University, Washington University, NCI, and Duke University were annotated by a group of 154 medical students and residents, approved by one of the neuroradiologists from ASFNR Annotator group or Super Annotator group, and underwent image and segmentation quality control evaluation (QC) by the study organizer and neuroradiologist (MSA). Details of changes in segmentations from student annotator group, to approver step, to final image and segmentation quality control step will be included in future manuscript.

## Discussion

Metastatic disease is an advanced stage of cancer, and when it spreads to the brain, it significantly shortens a patient’s life expectancy.^33–35^ Brain metastases can appear in several patterns: multiple small lesions throughout the brain, a solitary lesion with central necrosis and surrounding edema, or a mix of large and small lesions with and without prominent surrounding edema. The etiology of cancer that is metastatic to the brain and the pattern of metastatic disease are significant factors in the decision-making process for the type of therapy that is pursued for patients.^36,37^ It is critical to identify all metastatic lesions to the brain parenchyma, including those that are less than 5 mm in size, when diagnosing brain metastases. During the planning of targeted radiation therapy, individual lesions are identified and delineated on imaging studies and then these lesions are targeted for definitive treatment with radiation therapy.^37^ Even a single missed lesion can significantly change a patient’s treatment response, leading to reduced survival and an increased risk of recurrence.^38,39^ Therefore, it is important to have accurate detection capabilities for brain metastases to ensure effective management. The development of machine learning algorithms that can accurately detect and segment brain metastases, regardless of size, has the potential to significantly impact treatment response assessment in patients and improve workflow efficiencies in clinical practice.

Accurate detection and tracking of lesion volumes is also critical for patient prognosis. Prior literature demonstrated that gross total volume of metastatic disease within the brain has significant impact on patient survival and can play a role in the setting when treatment options that are considered are equivalent.^40,41^ Therefore, the ability to assess the gross total volume of brain metastases at diagnosis can significantly impact patient outcomes. In the post-treatment setting, being able to track the changes in lesion volumes and perilesional edema over time from the time of diagnosis and longitudinally is critical for decision making. Some of the current treatments for patients with brain metastatic disease include whole brain radiation therapy, immunotherapy, and molecularly targeted chemotherapy. Being able to follow lesions with these metrics as they develop radiation necrosis with different degrees of recurrent tumor can significantly change the type of therapy that would be implemented.

Multiple algorithms have been identified for segmentation of brain metastases over the last several years with the majority of them being deep learning algorithms demonstrating high Dice scores, predominantly above 0.9.^3,6,8–10,12,28,42–44^ One of the main limitations of available segmentation algorithms is their ability to detect lesions below the size of 5 mm. Accurate detection of small lesions is critically important in clinical practice because it is possible to miss small lesions due to human error, and missing these lesions can lead to substantial changes in patient outcomes. On the other hand, an AI tool with high sensitivity but low specificity, producing many false positives, would likely result in radiologists not using this tool in clinical practice due to the extra time it takes to eliminate false positives. Therefore, the development of a segmentation algorithm that can accurately detect and segment lesions and perilesional edema is critically needed in clinical practice. Using multi-institutional datasets is critical for developing a generalizable model that can be applied to various institutions. Therefore, the MICCAI ASNR BraTS-METS Challenge is an important initiative that has the potential to improve the treatment of patients with brain metastases in individual hospitals.

Previous attempts have been made to release publicly available datasets for brain metastases. However, these previously released datasets vary in their inclusion and exclusion criteria, imaging quality, and available MRI sequences, causing inconsistencies among them. Table # provides a summary of previously available datasets.

**Table:** 

Public dataset	Data publisher	Number of cases	Difference from BraTS datasets
NYUMets^2^	New York University (NYU)	1,429 patients	Contains post therapy cases Not all patients have images Many cases without brain metastasis
BrainMetShare^45^	Stanford University	156 patients	Not containing T2 sequence Contains post therapy cases Available in JPEG format
UCSF-BMSR^26^	University of California San Fransico (UCSF)	412 patients	Not containing T2 sequence Contains post therapy cases
Brain-TR-GammaKnife^46^	University of Mississippi (UMMC)	47 patients	Recently published
MOLAB^47^	University of Castilla-La Mancha	75 patients	Contains post therapy cases Recently published

One of the major limitations of building large open science datasets is the concern for patient privacy and ensuring the security of data. This can be addressed by establishing security settings, such as data de-identification using skull and face stripping from the MRI scan to remove facial features. Additionally, establishing a culture of sharing and educating institutions on the development of algorithms that are widely applicable across institutions is an important part of socializing the concept of Open Science. Defining a balance between the culture of Open Science and Patient Safety is critical and will lead toward development of future advancement in medical image analysis.

A major challenge in the BraTS-METS ASNR MICCAI Segmentation Challenge is preparation of the brain metastasis datasets with expert approved lesion annotations, a process of identifying and labeling lesions in medical images. Brain metastases are uniquely different from glioblastomas, meningiomas, and other brain tumors because they can vary widely in size and number, as well as in the amount of peritumoral edema and necrosis. This means that the time it takes to annotate a single study can range from 15 minutes to several hours, depending on the number of lesions, which can range from 1 to over 100. Therefore, we instituted a novel concept for data annotation that incorporates education on imaging of brain metastases, basic MR imaging physics, and concepts of open science as an educational series for annotators. This is a novel concept in dataset annotation because it focuses on the educational value that is extracted by annotating the images, rather than the exhaustive hours it takes to create a well-curated and annotated dataset. This concept utilizes the idea of deliberate learning in education, which involves focused learning of a topic by structured understanding and re-enforcement of all the details of the topic. In the setting of brain metastasis imaging, student annotators learn all the imaging appearances of brain metastases by performing the annotations and forcing themselves to decide on the annotation. They reinforce their learning by attending weekly hands-on sessions with leaders in brain tumor imaging, where they can ask questions that they developed during the annotation process. They also participate in a structured curriculum that includes understanding the MR brain metastasis imaging protocol, the physics behind individual sequences, and the imaging appearance of different disease entities that are found in brain metastases, such as microvascular white matter damage, microbleeds, and different stages of hemorrhage. This educational effort provides a focused time with exposure to experts to learn all types of presentations of brain metastases and essentially creates a training set for students to understand the imaging appearance of brain metastases.

There are multiple limitations to our approach. One of them is that the contributed datasets are heterogeneous. As a result, many cases have been excluded from the analysis because they contain resection cavities, post-treatment changes, or do not have brain parenchymal metastases. Also, some of the datasets did not have appropriate skull stripping, which can lead to portions of metastases being accidentally removed by the skull stripping software or not being detected at all. Another source of heterogeneity was due to differences in data acquisition, patient motion, protocols, slice thickness, and contrast injection timing which can lead to misregistration of images on different sequences. These limitations contribute to the heterogeneity of data, which can have both positive and negative implications. On one hand, it can pose challenges for developing a uniform segmentation algorithm. On the other hand, it can also provide a diverse range of data that can enhance and generalize algorithm development. We are looking forward to the results of the challenge to learn how data heterogeneity contributes to the results of individual algorithms. Furthermore, skull stripping can make it difficult to describe and differentiate dural-based lesions, such as metastases and meningiomas. Skull stripping can also remove some of the brain parenchyma, making it difficult to identify metastases that are commonly occurring at the gray-white junction. Skull stripping also limits the evaluation of osseous metastases to the calvarium. Finally, transferring cases from different annotation software can degrade mask quality, as masks can be altered when registered to different atlas spaces, which we addressed by registering the images to the common SRI24 atlas.

Another major limitation of the current challenge is that it is very complex to annotate ground truth data for brain metastases. This is because brain metastases are typically small and there can be many of them in a single scan. During the annotation process, we identified many instances where segmentation needed to be improved. For example, the Yale Brain Metastasis dataset was segmented by a medical student and then refined by two neuroradiologists. However, when the dataset and segmentations were transferred to the BraTS challenge and reprocessed and re-oriented with a new atlas, many of the segmentations had to be revised, especially the small lesions that appeared on only one or two image slices. Additionally, new metastases, necrotic portion of the tumor, and peritumoral edema on FLAIR images were identified during the revision process. This highlights the fact that many iterations of ground truth segmentation are often required when annotating databases for research purposes and that standardization of the annotation labels to more efficiently export segmentations is needed. We recommend that challenge participants reach out to the organizers if they do not agree with the segmentation or if some of the segmentations are not correlating with their algorithm output. The organizers will revise the segmentations and update the data as needed.

## Figures and Tables

**Table 1:** Datasets included in the first release of the challenge on June 1, 2023.

Dataset source	Final without correction	Final with correction	Excluded – No lesion or incomplete scan	Excluded-post treatment	Final included	Final excluded
NYU	107	57	46	22	164	68
NCI	21	11	1	0	32	1
Duke	14	10	0	0	24	0
WashU	16	12	0	1	28	1
Yale	44	36	0	0	80	0
Total Released June 1, 2023					328	
