# Supplementary material for: The Brain Tumor Segmentation - Metastases (BraTS-METS) Challenge 2023: Brain Metastasis Segmentation on Pre-treatment MRI
Source: ArXiv. 2024 Dec 9:arXiv:2306.00838v3. Originally published 2023 Jun 1. Preprint. [Version 3] (PMC10312806)
Supplement: Supplement 1 [file NIHPP2306.00838v3-supplement-1.pdf]

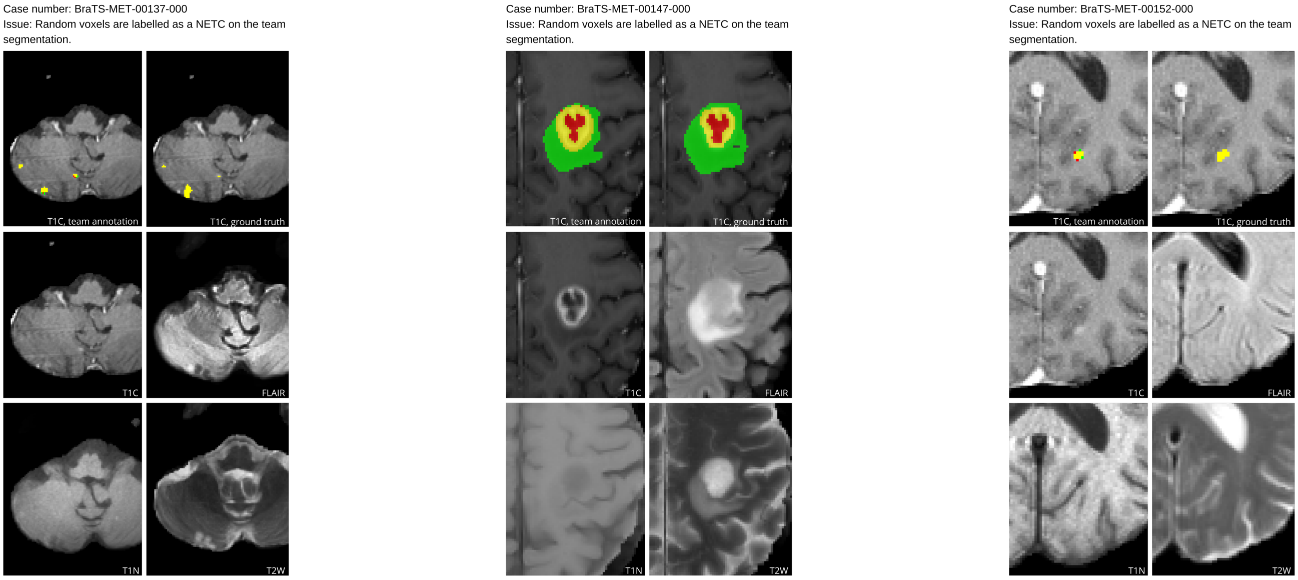

Figure 11: Supplementary: Examples of Random Voxels Predicted as Non-enhancing tumor core

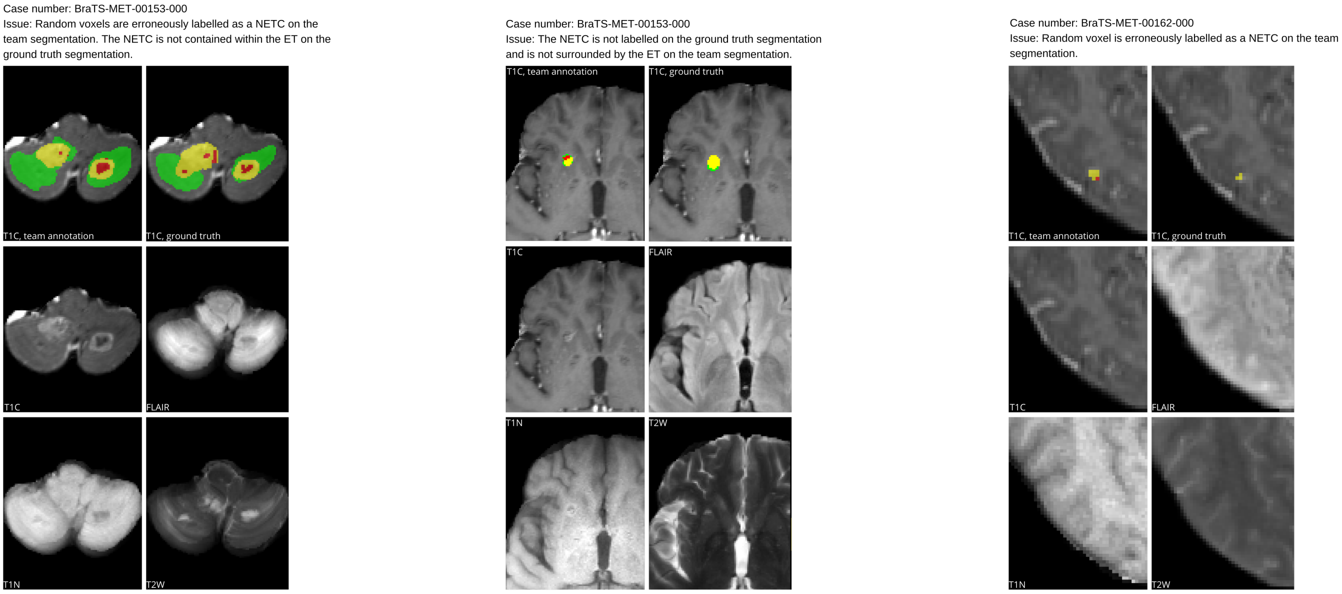

Figure 12: Supplementary: Examples of Random Voxels Predicted as Non-enhancing tumor core

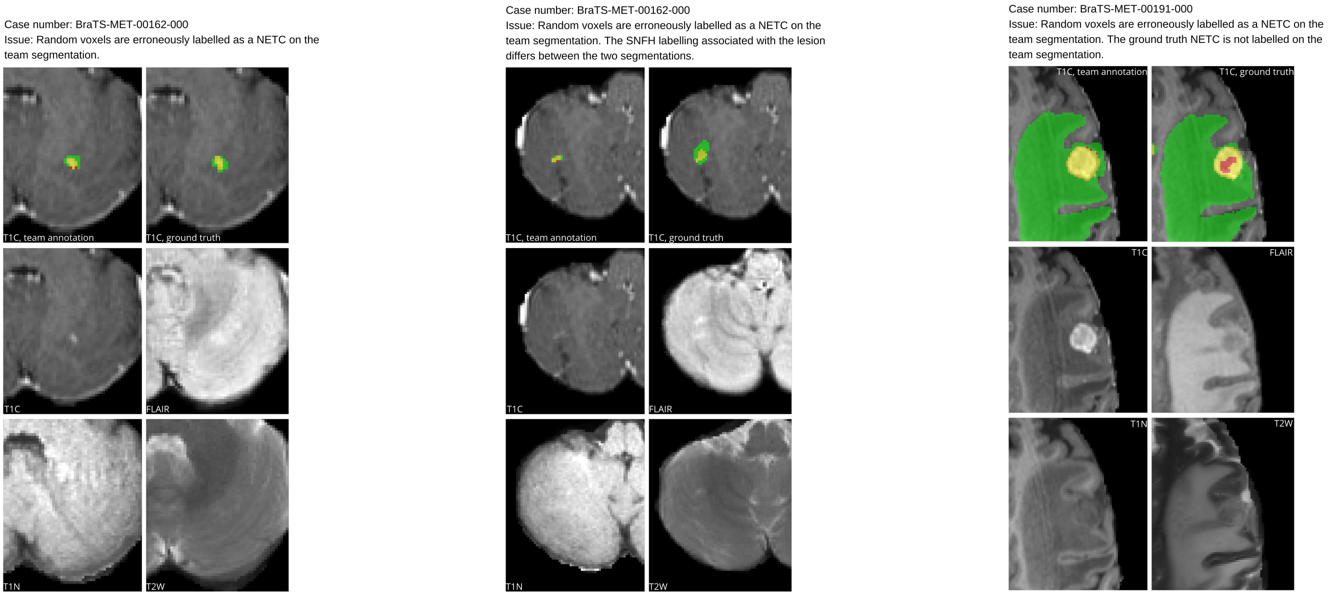

Figure 13: Supplementary: Examples of Random Voxels Predicted as Non-enhancing tumor core

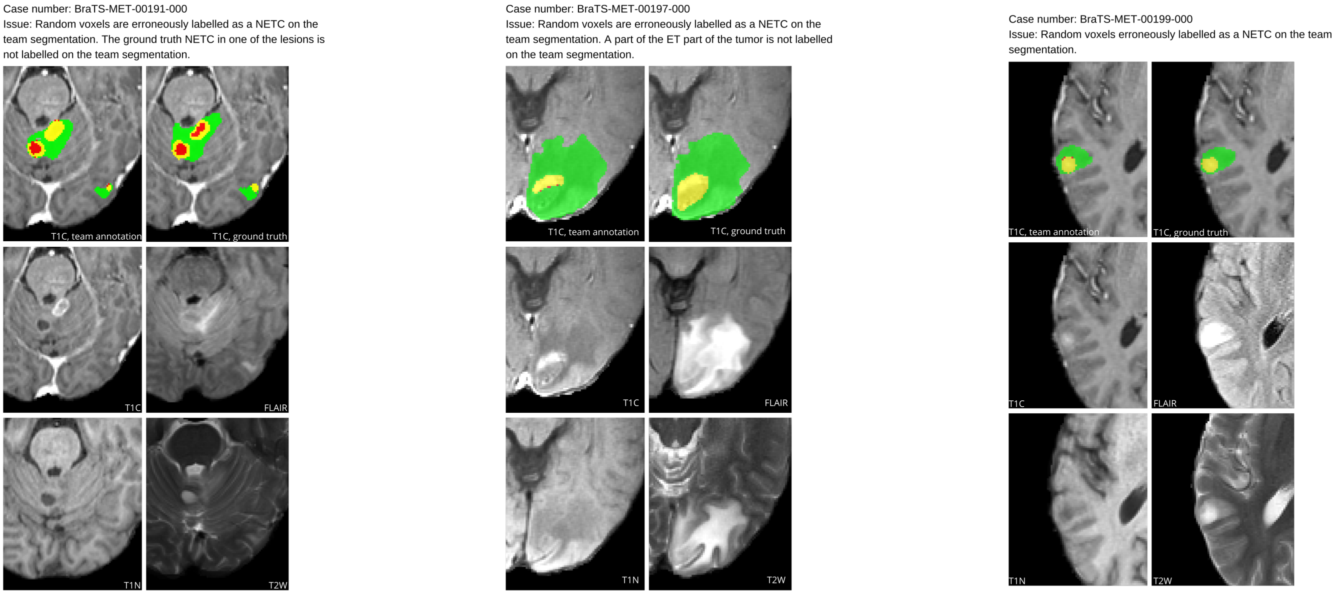

Figure 14: Supplementary: Examples of Random Voxels Predicted as Non-enhancing tumor core

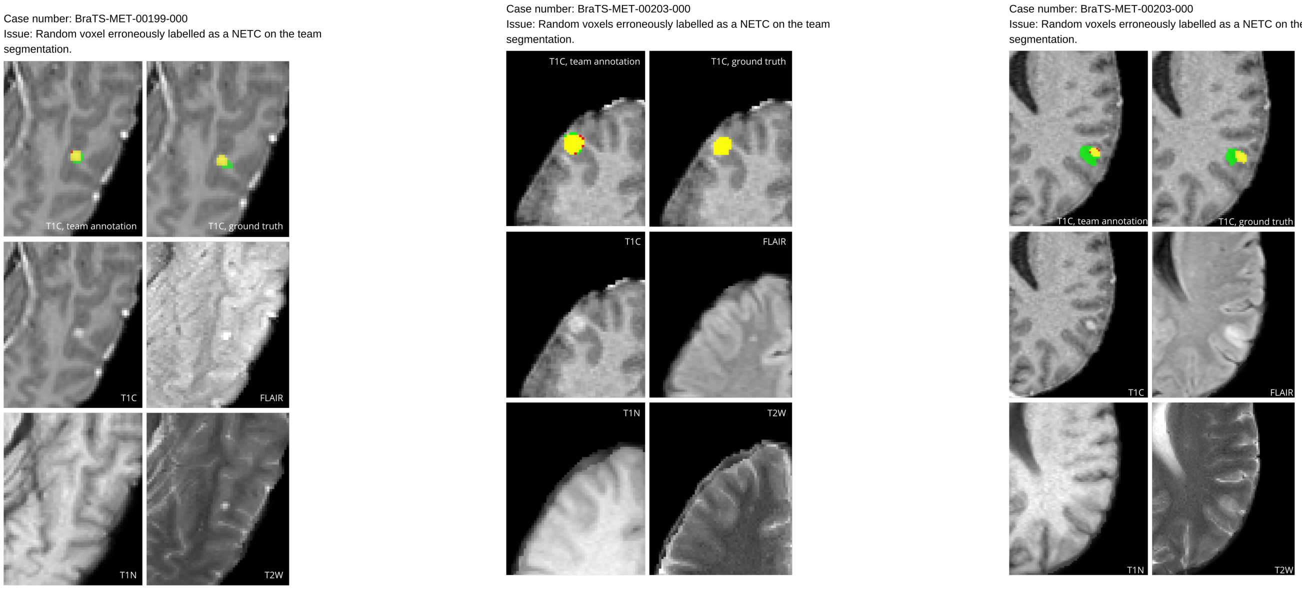

Figure 15: Supplementary: Examples of Random Voxels Predicted as Non-enhancing tumor core

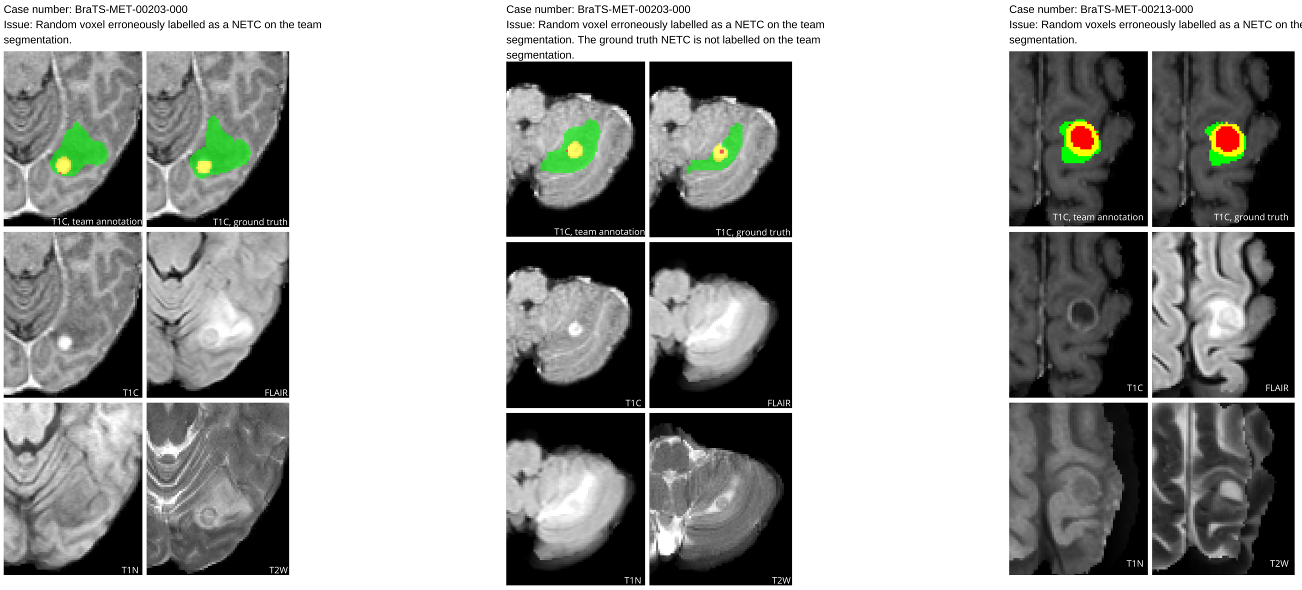

Figure 16: Supplementary: Examples of Random Voxels Predicted as Non-enhancing tumor core

Case number: BraTS-MET-00216-000
Issue: Random voxel erroneously labelled as a NETC on the team segmentation.

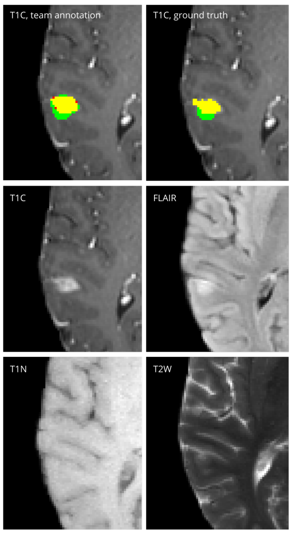

Case number: BraTS-MET-00221-000
Issue: Random voxel erroneously labelled as a NETC on the team segmentation. The NETC label is not fully contained within the ET borders on both the segmentations.

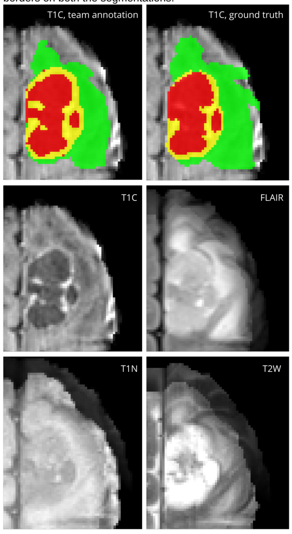

Case number: BraTS-MET-00252-000
Issue: Random voxel erroneously labelled as a NETC on the team segmentation.

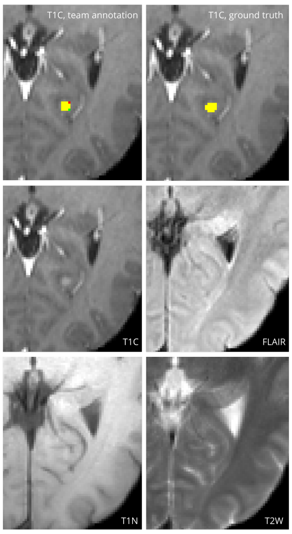

Figure 17: Supplementary: Examples of Random Voxels Predicted as Non-enhancing tumor core

Case number: BraTS-MET-00776-000
Issue: Random voxels erroneously labelled as a NETC. Part of the saggittal sinus was marked as part of the enhancing tumor.

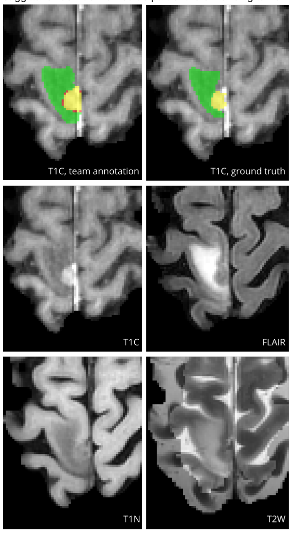

Case number: BraTS-MET-00276-000
Issue: Random voxels erroneously labelled as a NETC.

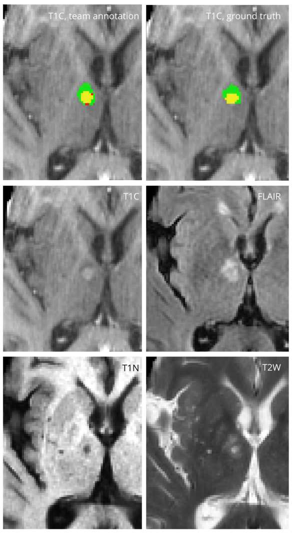

Case number: BraTS-MET-00276-000
Issue: Random voxels erroneously labelled as a NETC on the team segmentation. The cranialmost part of the met was not segmented on the ground truth.

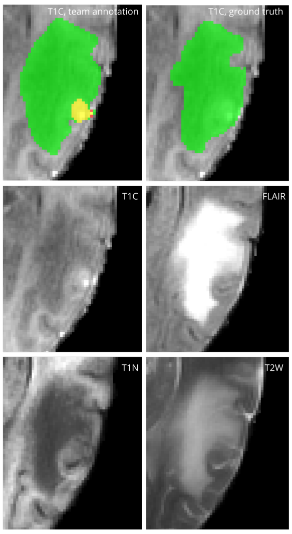

Figure 18: Supplementary: Examples of Random Voxels Predicted as Non-enhancing tumor core

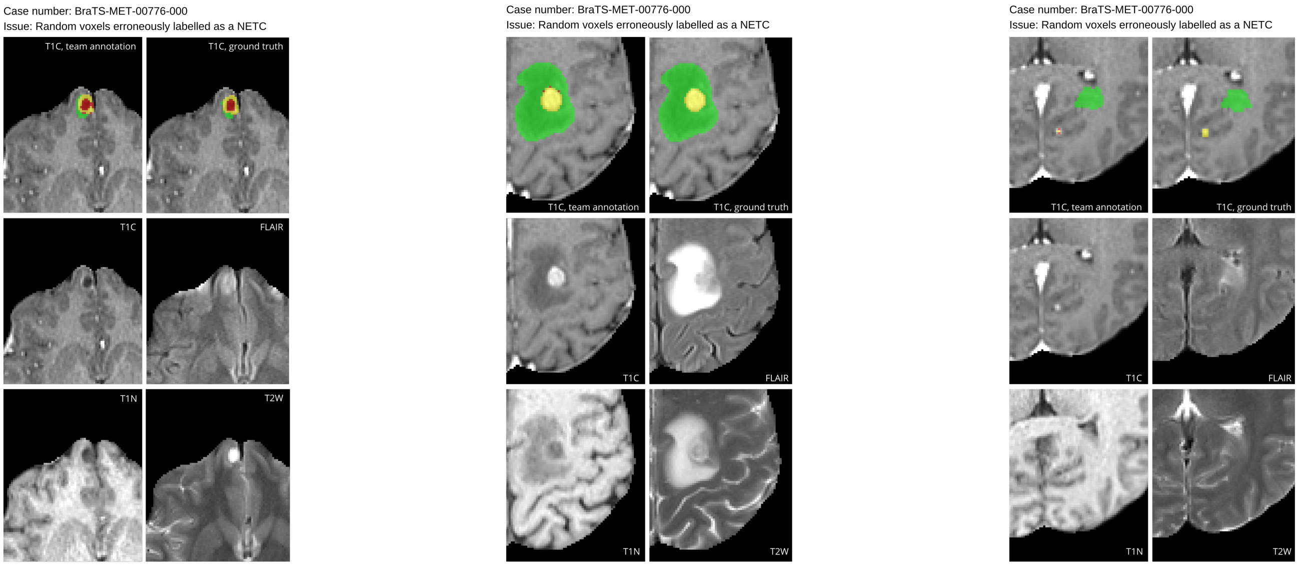

Figure 19: Supplementary: Examples of Random Voxels Predicted as Non-enhancing tumor core

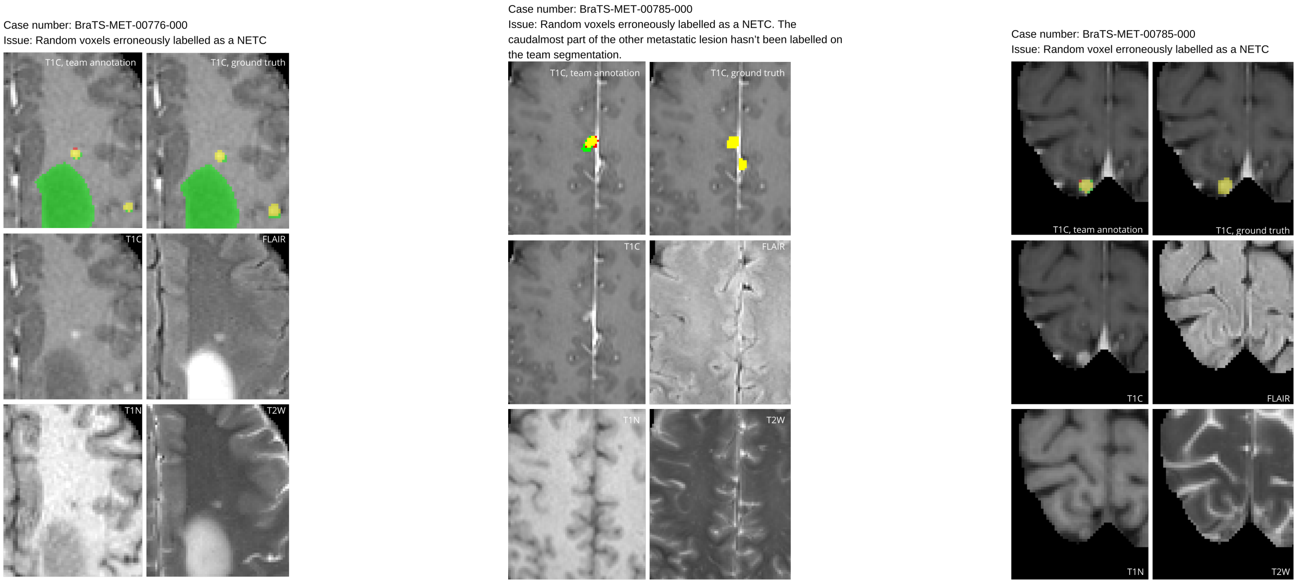

Figure 20: Supplementary: Examples of Random Voxels Predicted as Non-enhancing tumor core

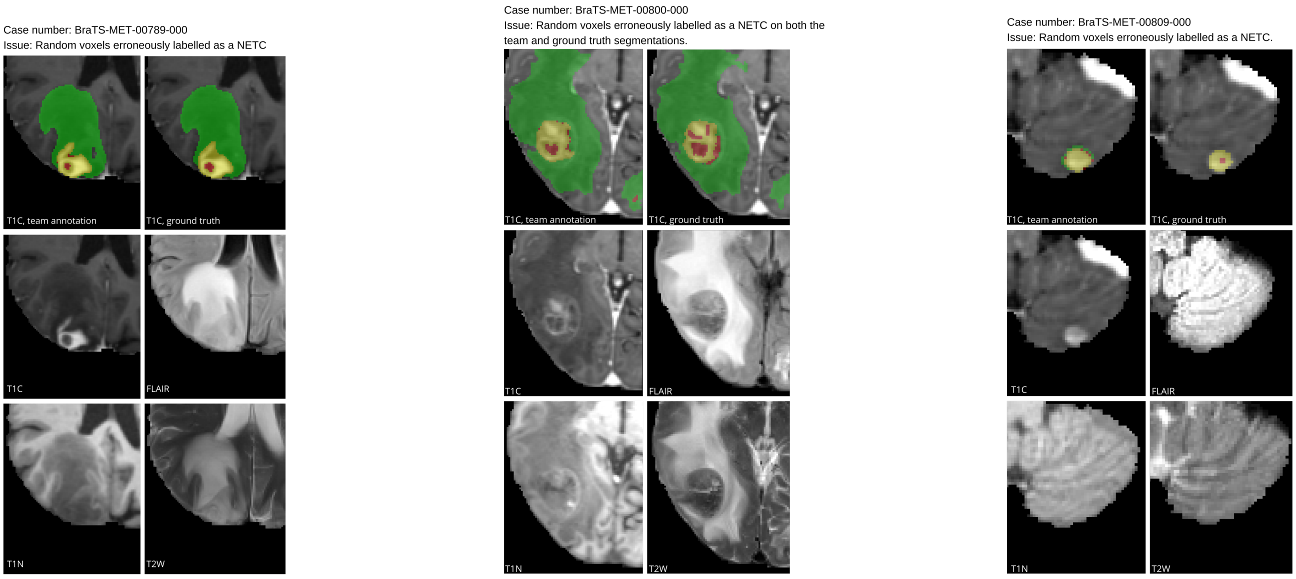

Figure 21: Supplementary: Examples of Random Voxels Predicted as Non-enhancing tumor core

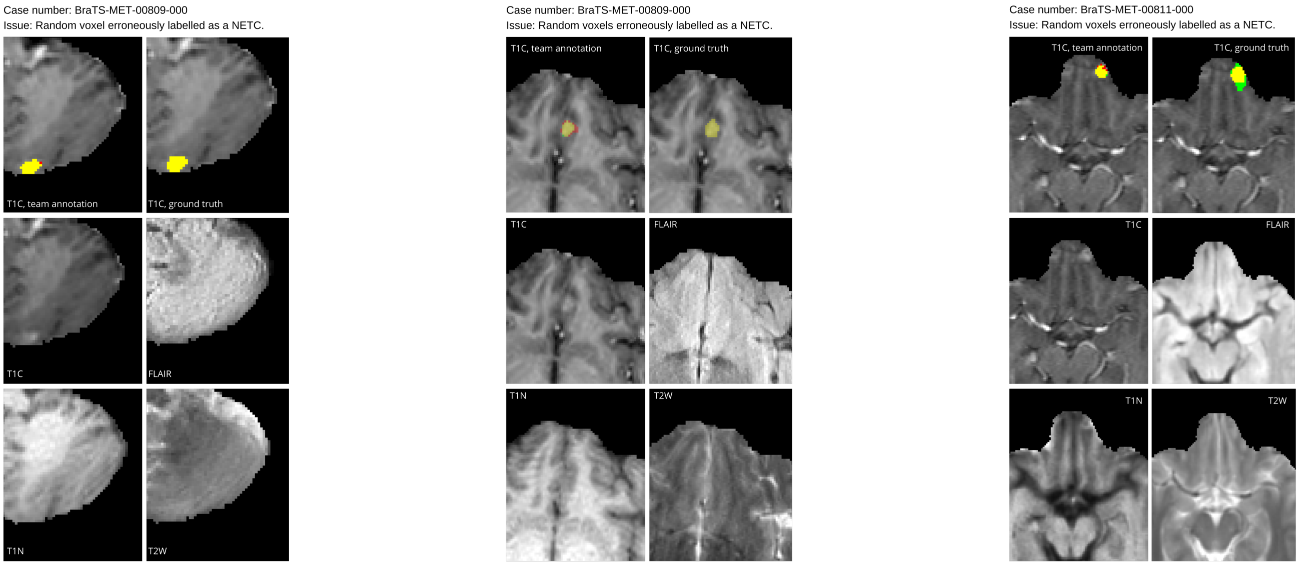

Figure 22: Supplementary: Examples of Random Voxels Predicted as Non-enhancing tumor core

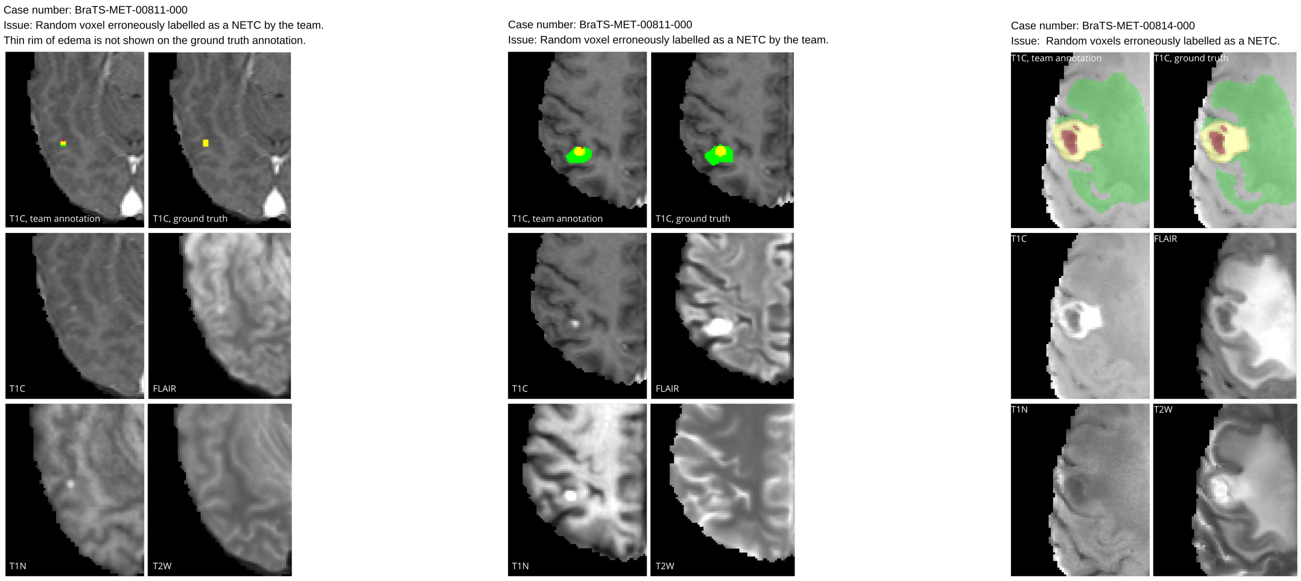

Figure 23: Supplementary: Examples of Random Voxels Predicted as Non-enhancing tumor core

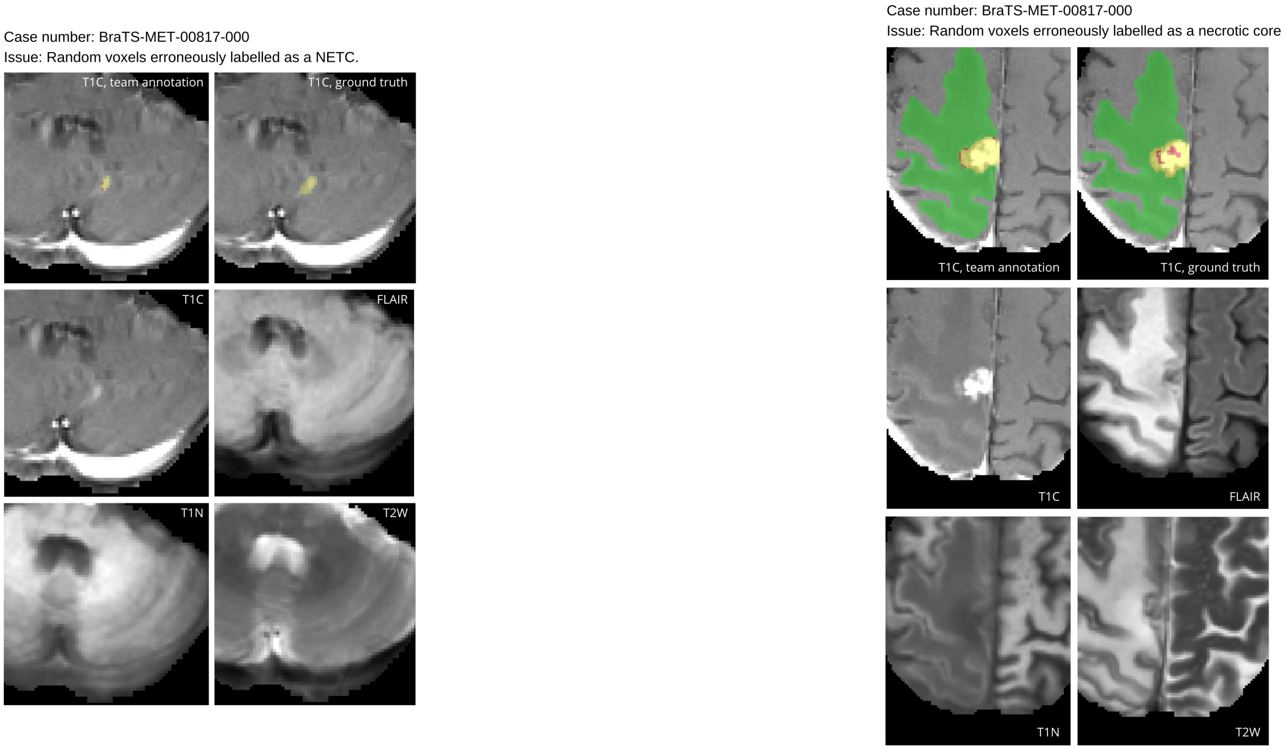

Figure 24: Supplementary: Examples of Random Voxels Predicted as Non-enhancing tumor core

Case number: BraTS-MET-00137-000
Issue: There is one metastasis missed on the team segmentation. There are some voxels, associated with another lesion, marked as NETC on the team segmentation, but not on the ground truth one.

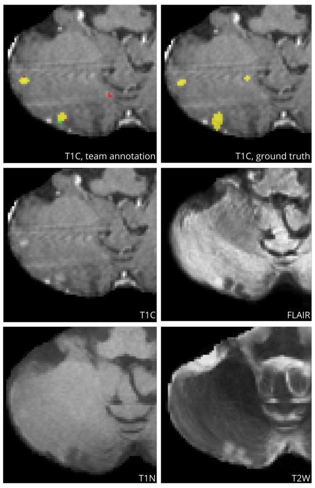

Case number: BraTS-MET-00137-000
Issue: There is one metastasis missed on the ground truth segmentation.

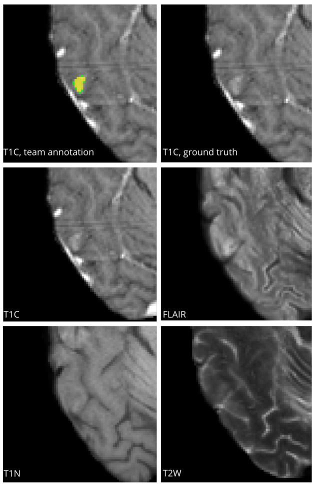

Case number: BraTS-MET-00144-000
Issue: Three metastases were not labelled as an ET on the team segmentation.

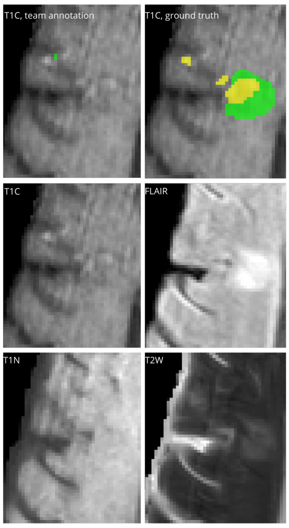

Figure 25: Supplementary: Pitfall Cases

Case number: BraTS-MET-00144-000
Issue: Part of a vessel was marked as CE lesion on the ground truth segmentation.

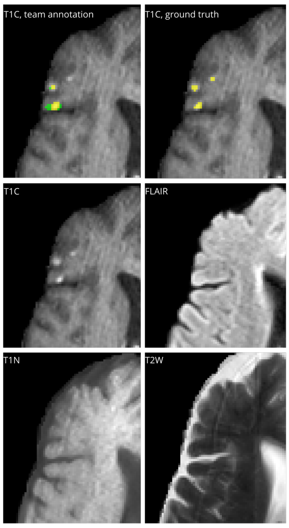

Case number: BraTS-MET-00144-000
Issue: Metastasis was missed on the team segmentation.

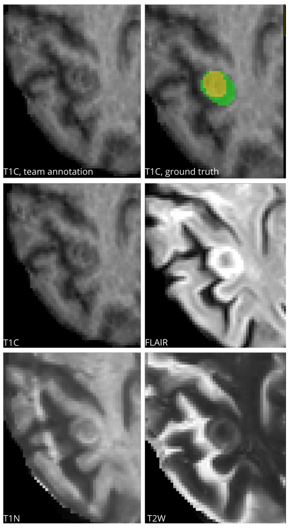

Case number: BraTS-MET-00144-000
Issue: Metastasis was missed on the team segmentation. FLAIR hyperintensity was labelled without being in direct association with the lesion

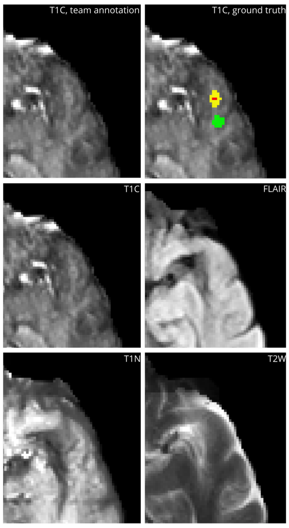

Figure 26: Supplementary: Pitfall Cases

Case number: BraTS-MET-00147-000
Issue: Metastasis was missed on the team segmentation.

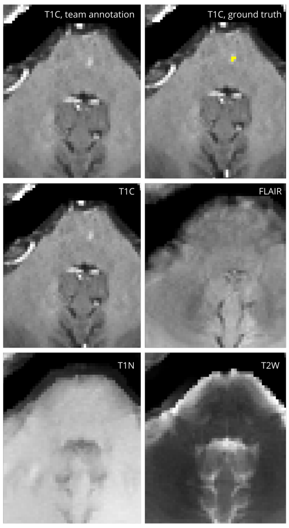

Case number: BraTS-MET-00148-000
Issue: Metastasis was missed on the team segmentation.

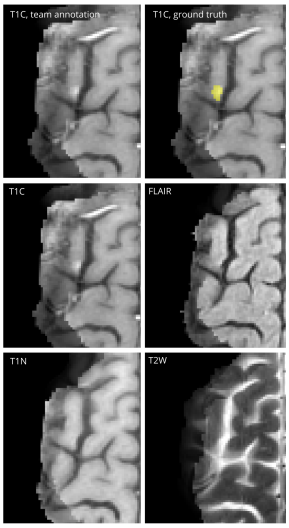

Case number: BraTS-MET-00148-000
Issue: Metastasis was missed on the team segmentation.

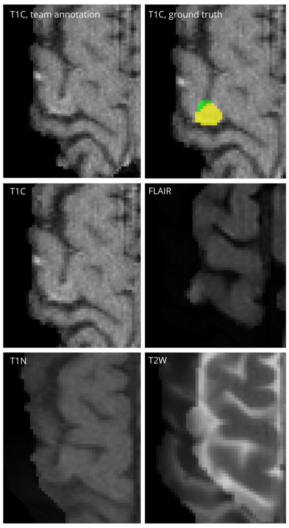

Figure 27: Supplementary: Pitfall Cases

Case number: BraTS-MET-00152-000
Issue: A vessel was erroneously marked as a small met on the team segmentation.

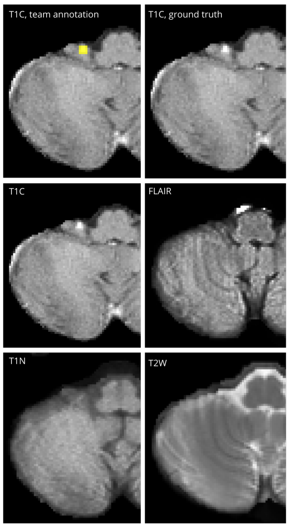

Case number: BraTS-MET-00162-000
Issue: Metastasis was missed on the ground truth segmentation.

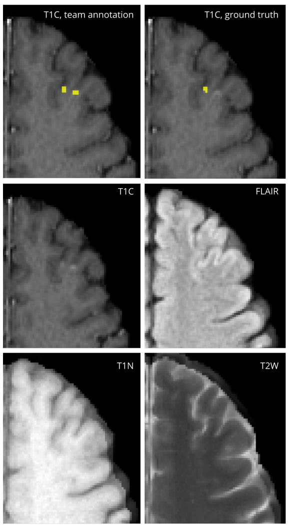

Case number: BraTS-MET-00162-000
Issue: Metastasis was missed on the ground truth and marked as NETC on the team segmentation.

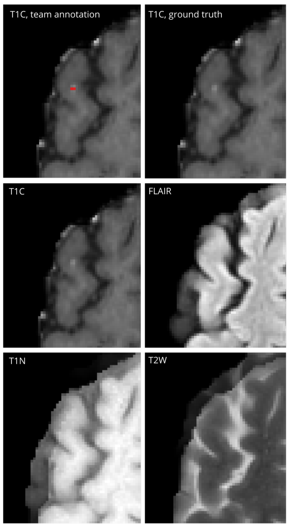

Figure 28: Supplementary: Pitfall Cases

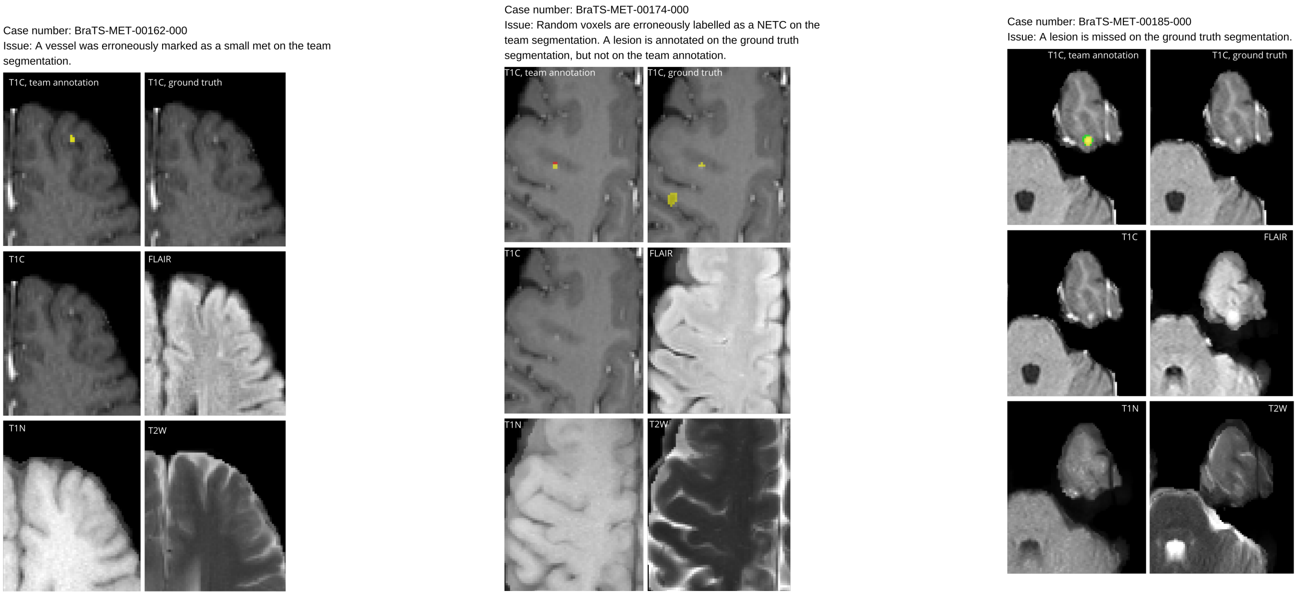

Figure 29: Supplementary: Pitfall Cases

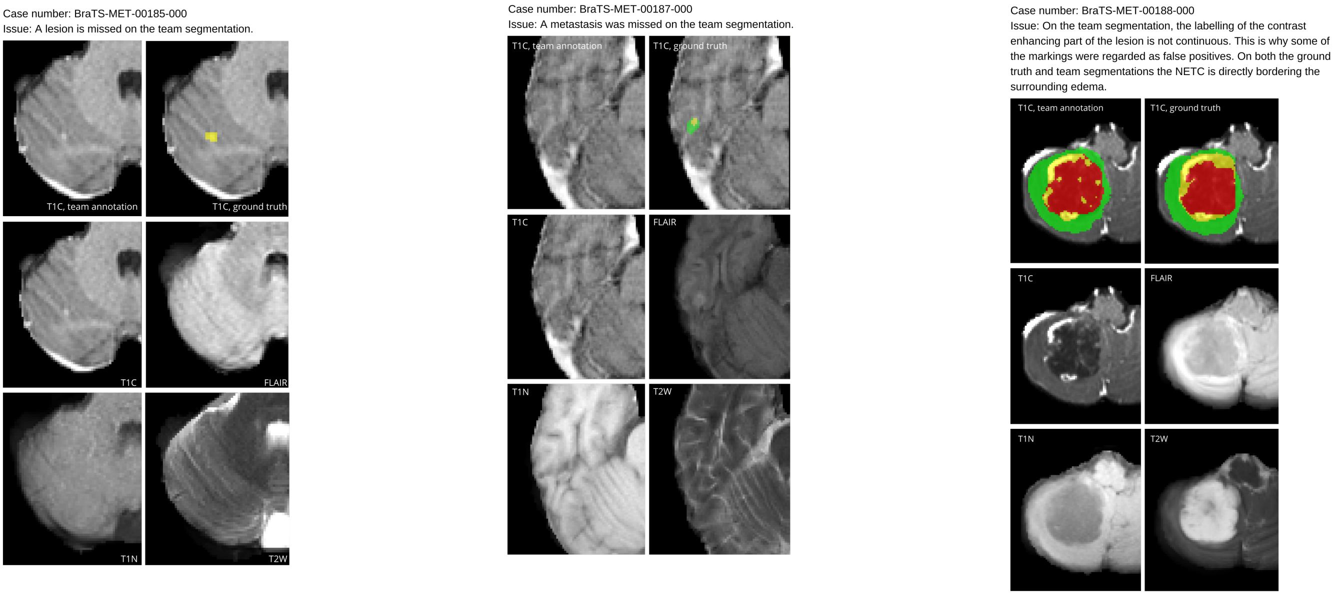

Figure 30: Supplementary: Pitfall Cases

Case number: BraTS-MET-00188-000
Issue: There was a metastatic lesion missed on the team segmentation.

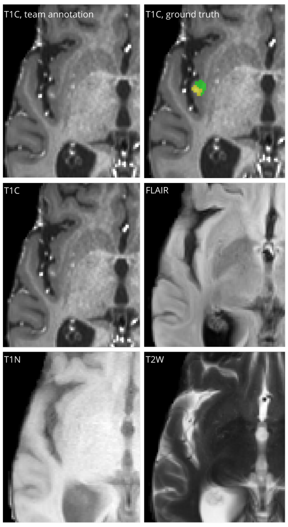

Case number: BraTS-MET-00191-000
Issue: There was a metastatic lesion missed on the team segmentation.

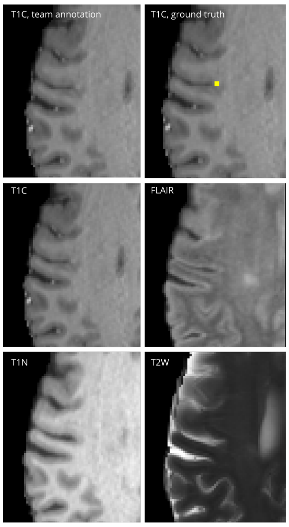

Case number: BraTS-MET-00191-000
Issue: There was a metastatic lesion missed on the team segmentation.

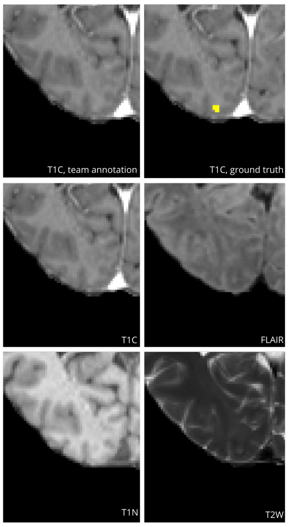

Figure 31: Supplementary: Pitfall Cases

Case number: BraTS-MET-00191-000
Issue: There was a metastatic lesion missed on the team segmentation.

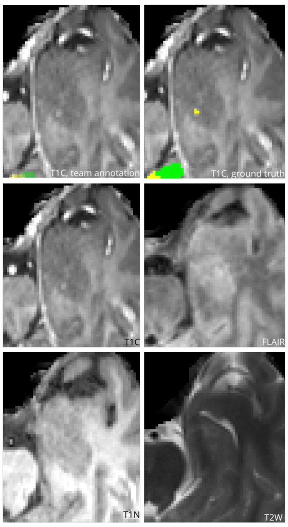

Case number: BraTS-MET-00197-000
Issue: Metastasis was missed on the team segmentation.

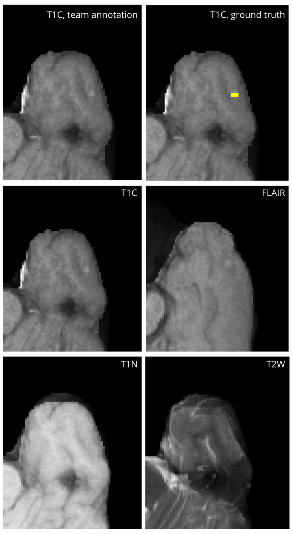

Case number: BraTS-MET-00197-000
Issue: Metastasis was missed on the team segmentation.

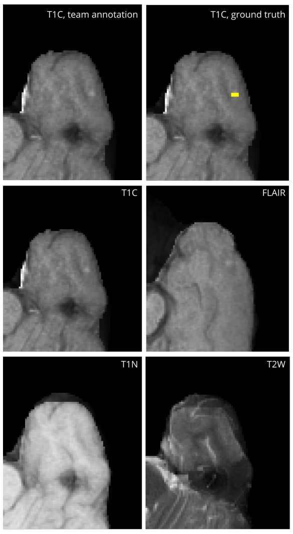

Figure 32: Supplementary: Pitfall Cases

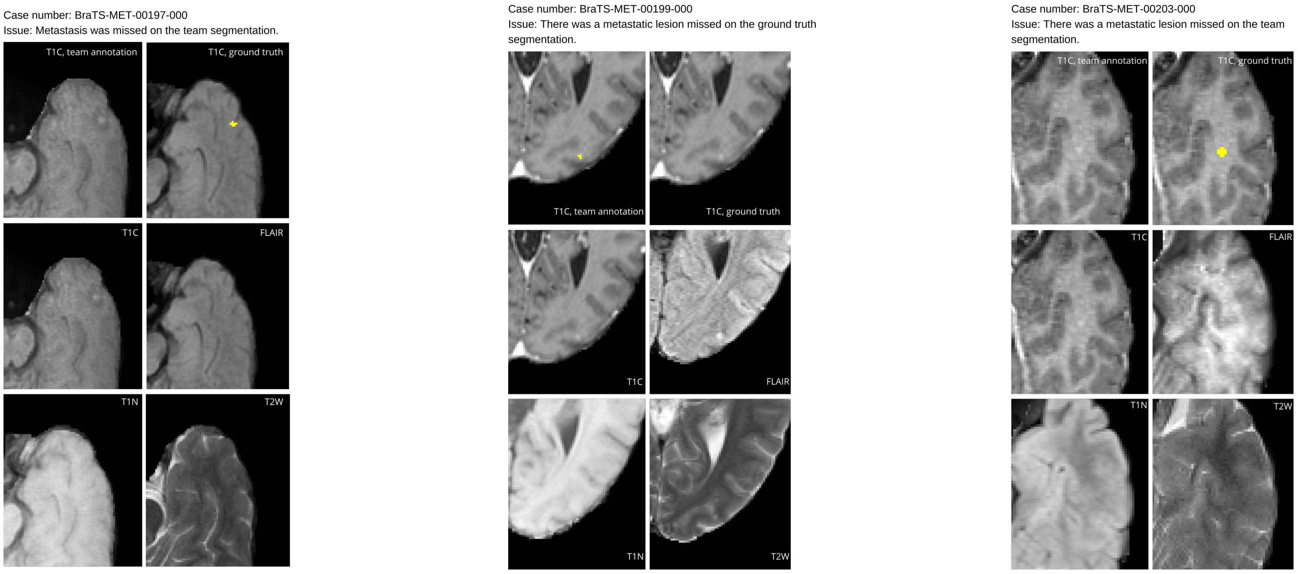

Figure 33: Supplementary: Pitfall Cases

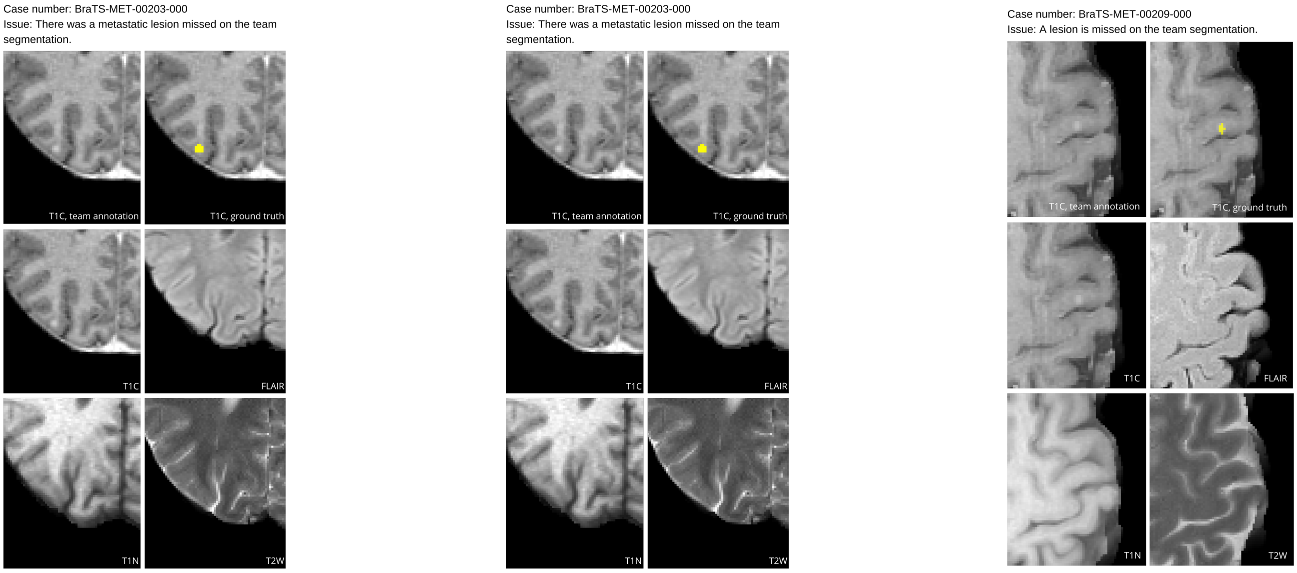

Figure 34: Supplementary: Pitfall Cases

Case number: BraTS-MET-00209-000
Issue: What looks to be continuous with a vessel has been marked as a metastatic lesion on the team segmentation .

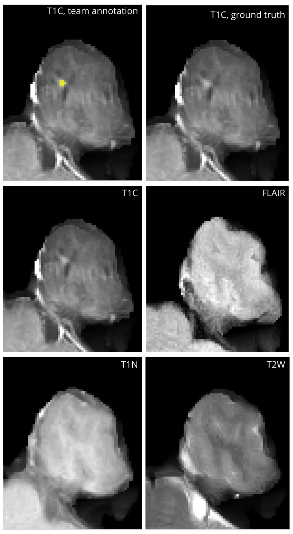

Case number: BraTS-MET-00209-000
Issue: What looks to be continuous with a vessel has been marked as a metastatic lesion on the ground truth segmentation .

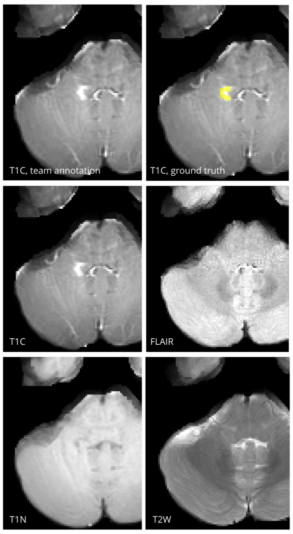

Case number: BraTS-MET-00213-000
Issue: What looks like an M2 aneurysm has been marked as a met on the team segmentation.

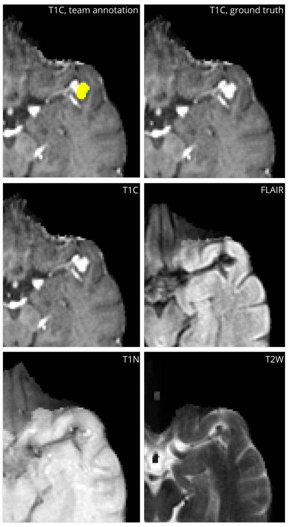

Figure 35: Supplementary: Pitfall Cases

Case number: BraTS-MET-00216-000
Issue: Metastatic lesion was missed on the team segmentation.

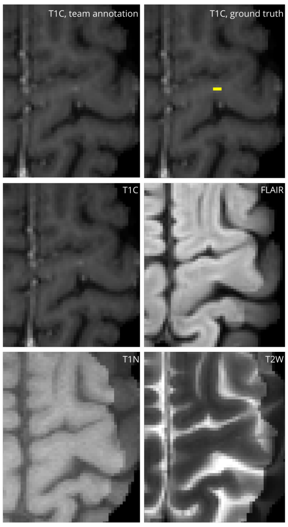

Case number: BraTS-MET-00221-000
Issue: A vessel was marked as a met on the ground truth segmentation.

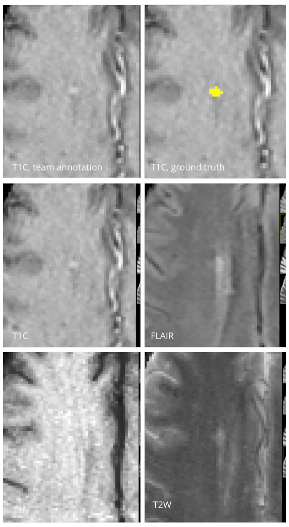

Case number: BraTS-MET-00239-000
Issue: A metastasis was missed on the team segmentation.

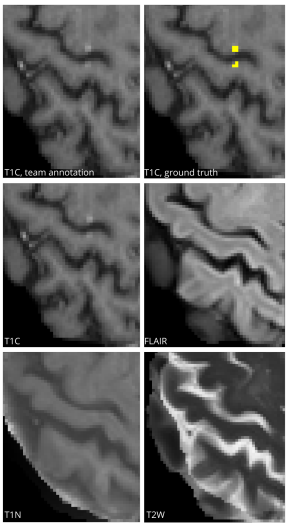

Figure 36: Supplementary: Pitfall Cases

Case number: BraTS-MET-00252-000
Issue: A metastasis was not labelled on the team segmentation.

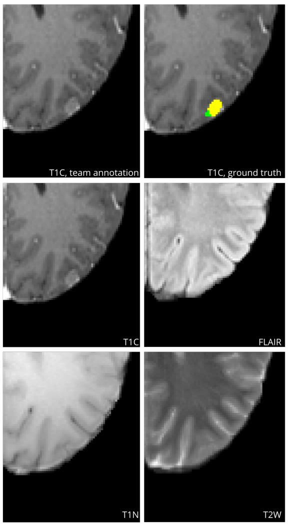

Case number: BraTS-MET-00276-000
Issue: A vessel was marked as a small met on the team segmentation.

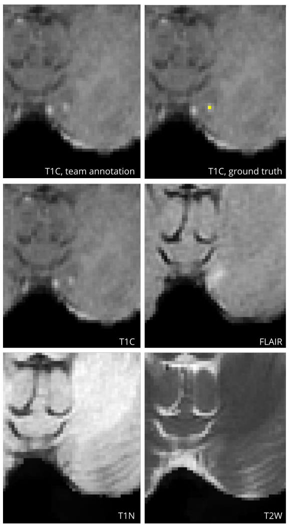

Case number: BraTS-MET-00776-000
Issue: A small metastasis was marked as SNFH

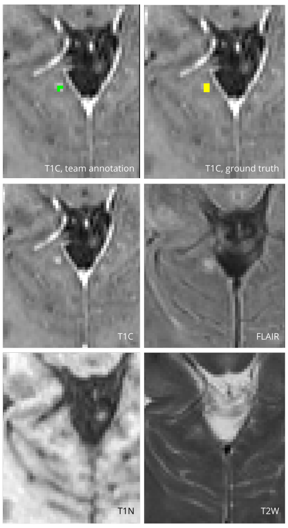

Figure 37: Supplementary: Pitfall Cases

Case number: BraTS-MET-00789-000
Issue: What looks like a part of a vessel has been marked as a small met on the ground truth segmentation.

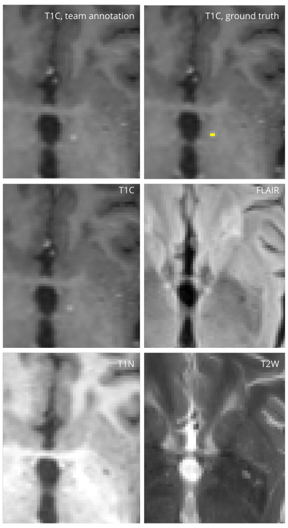

Case number: BraTS-MET-00800-000
Issue: Part of the signal from transverse-sigmoid sinus junction has been marked as ET on the ground truth segmentation.

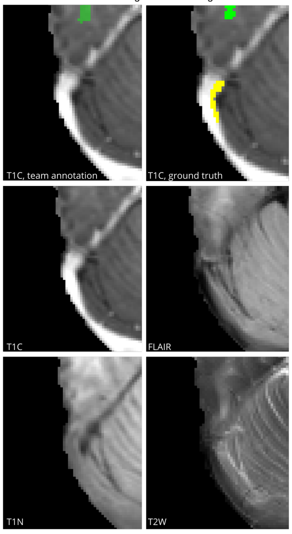

Case number: BraTS-MET-00809-000
Issues:
-One part of a metastatic lesion is labelled as edema on the team annotation (A) and another part of it is totally missed (B).
-On the ground truth image, some of the necrotic core is labelled in a way that suggests it is not contained within the ET part of the tumor (A). On another slice a couple of voxels within the lesion are not assigned neither to NETC, nor to ET (B).

A.

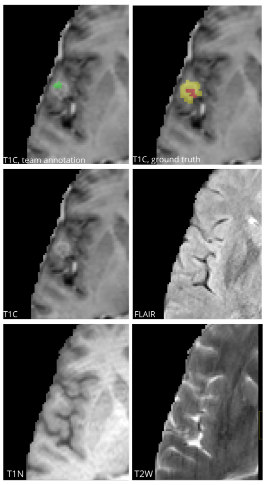

Figure 38: Supplementary: Pitfall Cases

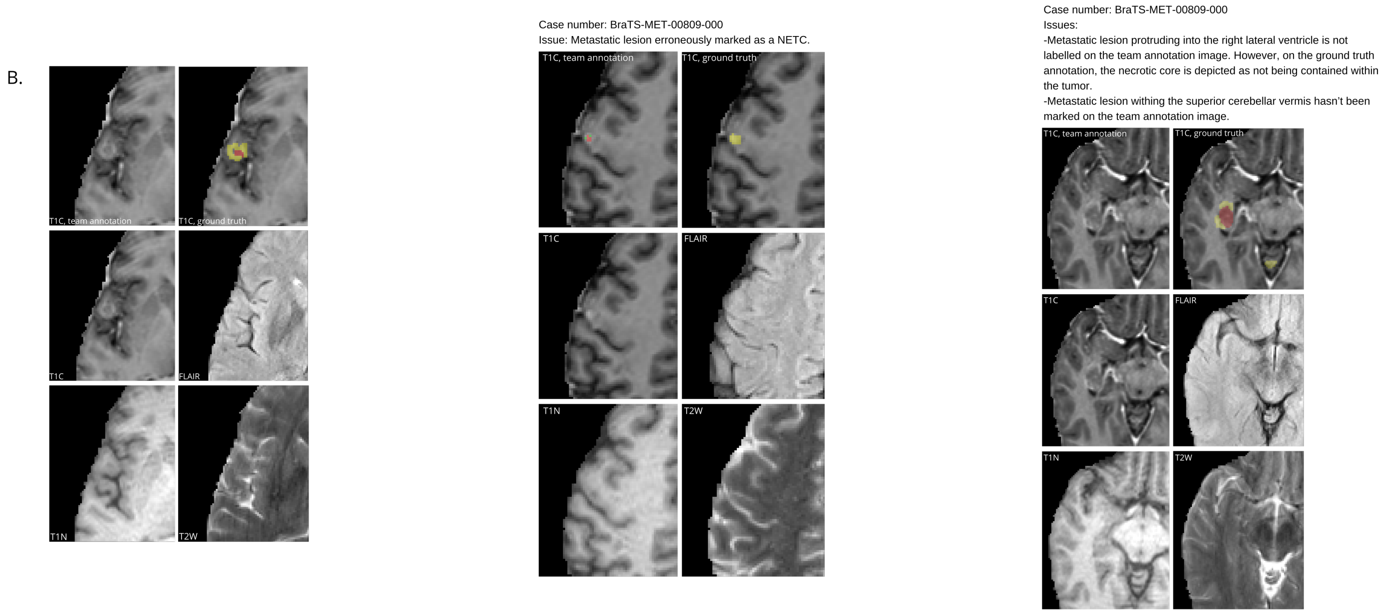

Figure 39: Supplementary: Pitfall Cases

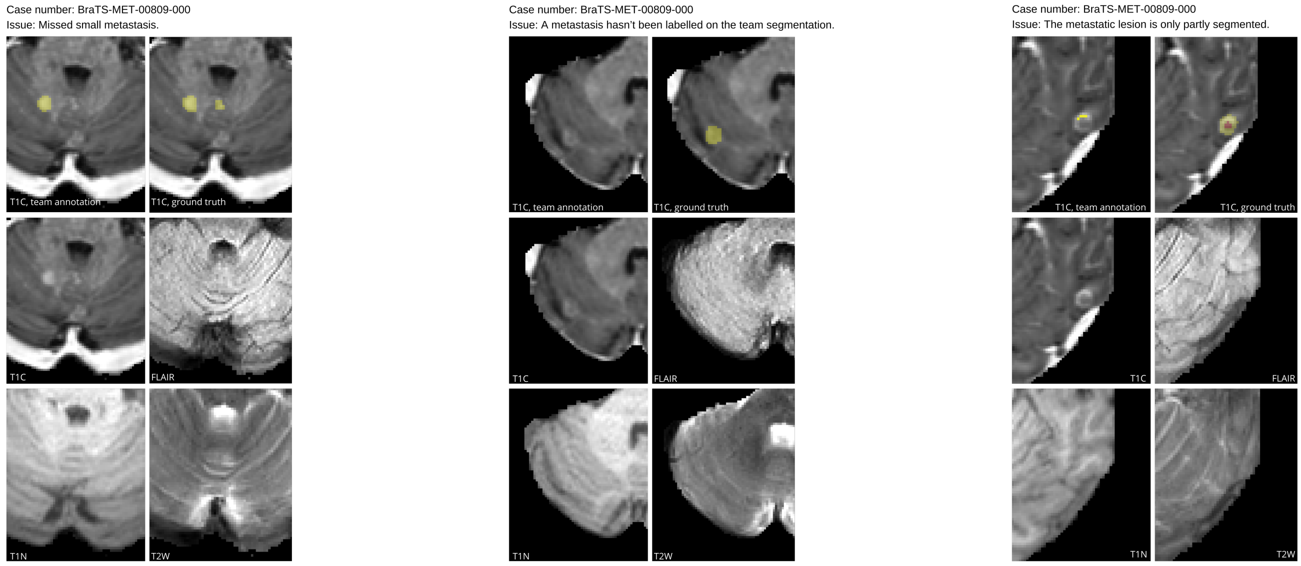

Figure 40: Supplementary: Pitfall Cases

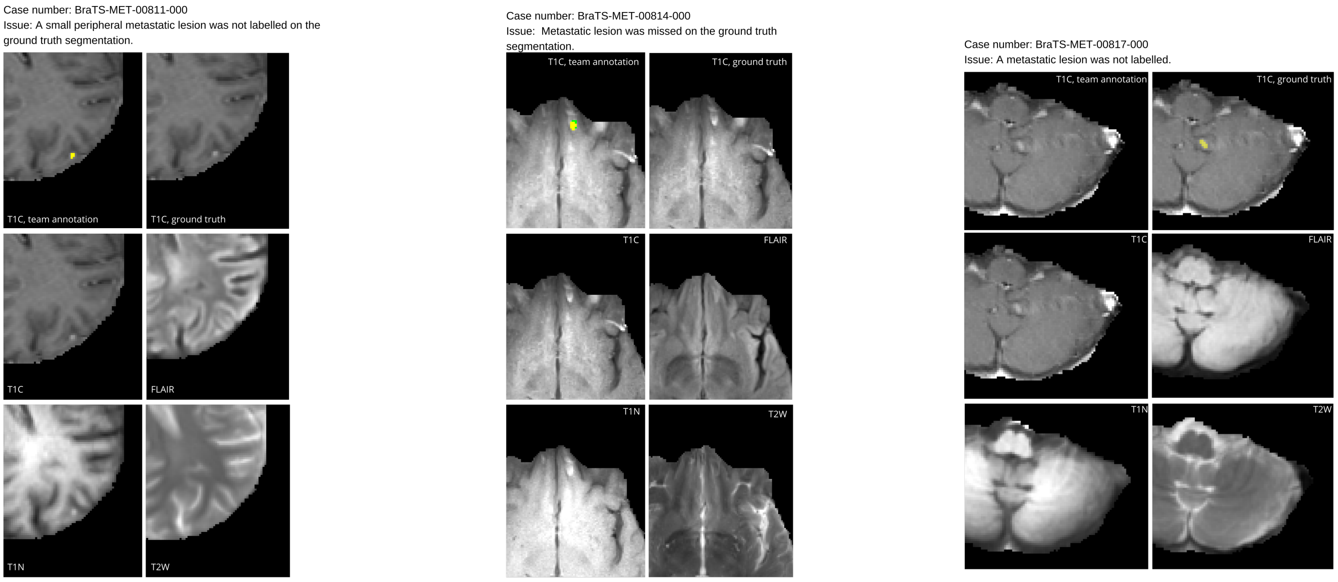

Figure 41: Supplementary: Pitfall Cases

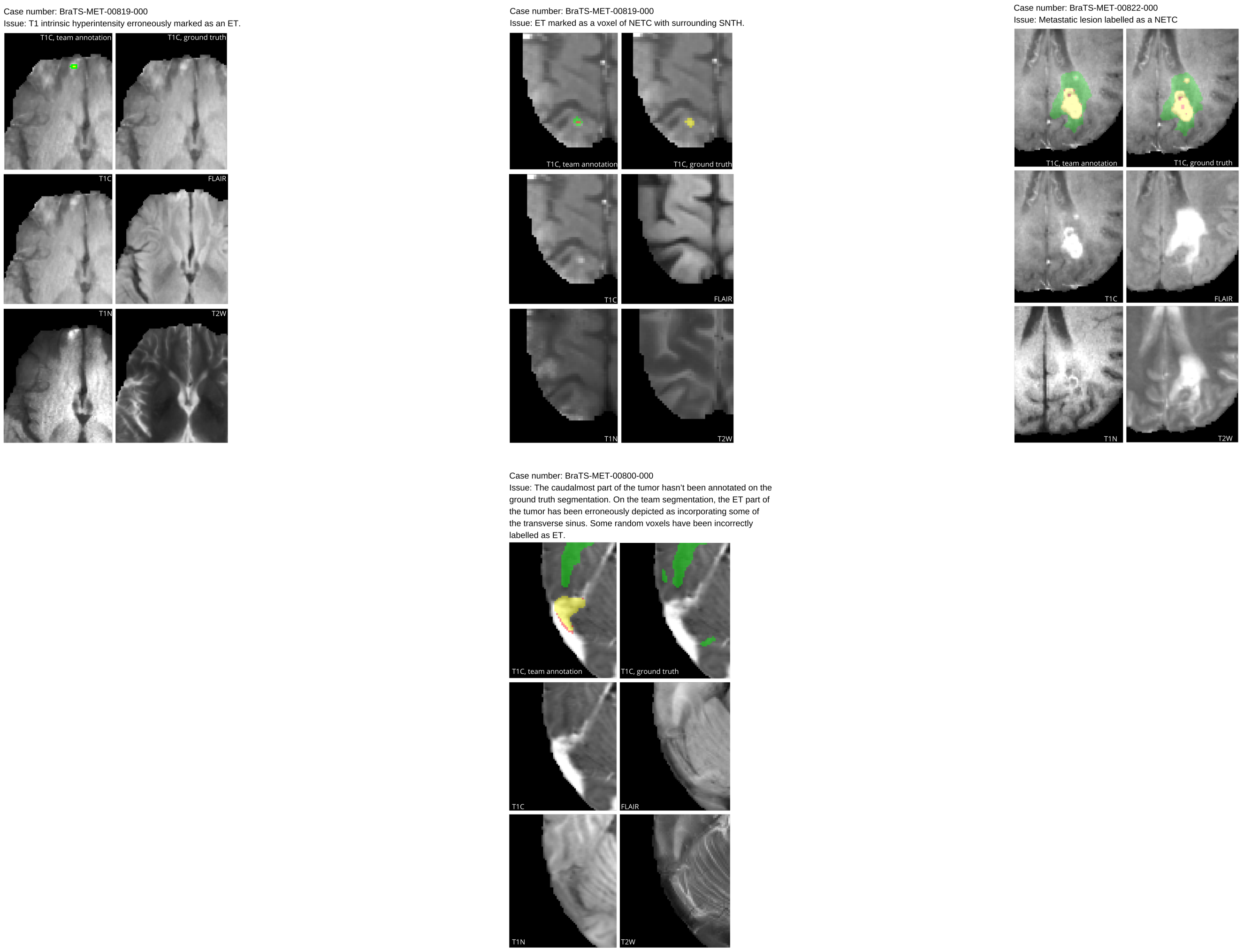

Figure 42: Supplementary: Pitfall Cases
