# Supplementary material for: The Brain Tumor Segmentation - Metastases (BraTS-METS) Challenge 2023: Brain Metastasis Segmentation on Pre-treatment MRI
Source: ArXiv. 2024 Jun 17:arXiv:2306.00838v2. Originally published 2023 Jun 1. Preprint. [Version 2] (PMC10312806)
Supplement: Supplementary file 1 [file NIHPP2306.00838v2-supplement-1.pdf]

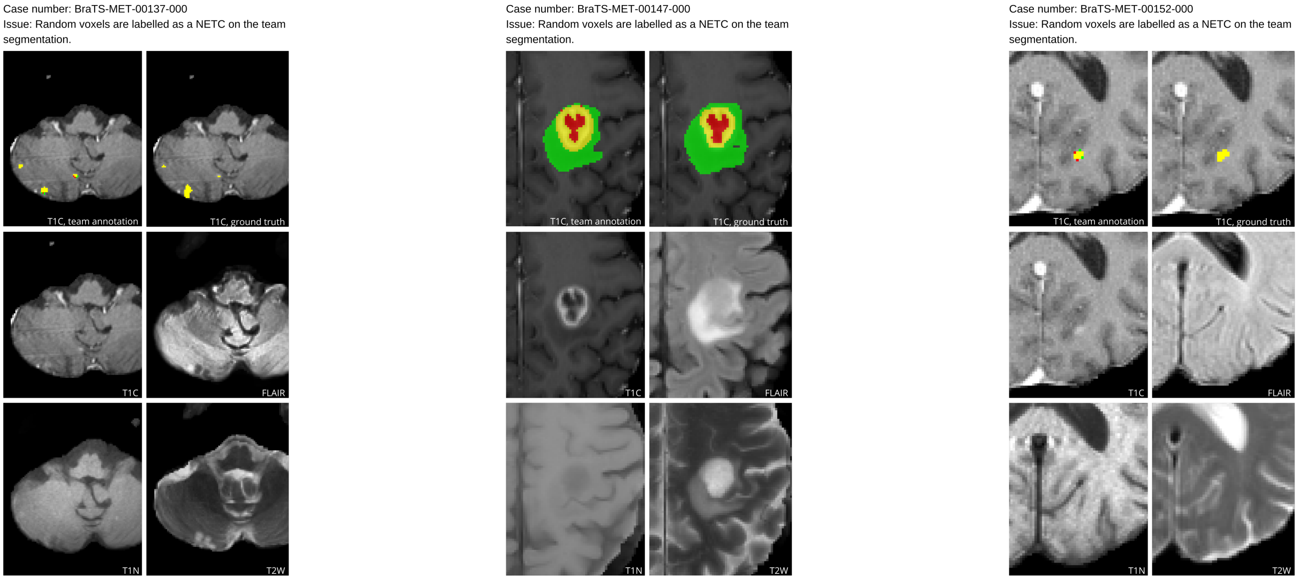

Figure 11: Supplementary: Examples of Random Voxels Predicted as Non-enhancing tumor core

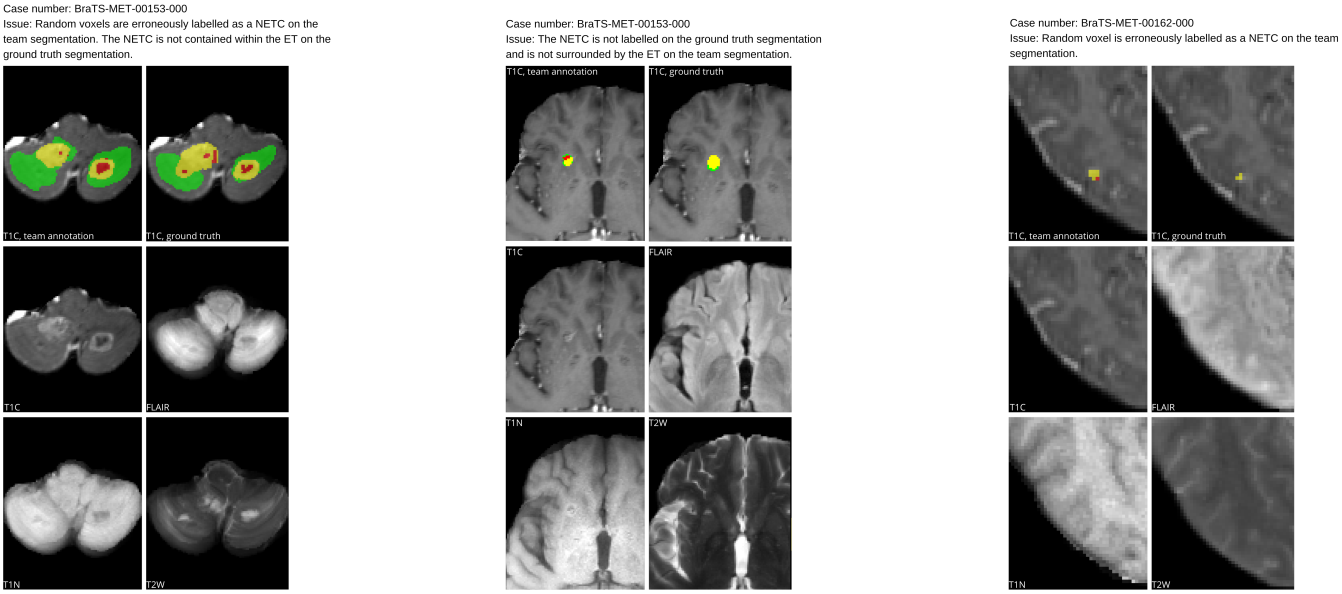

Figure 12: Supplementary: Examples of Random Voxels Predicted as Non-enhancing tumor core

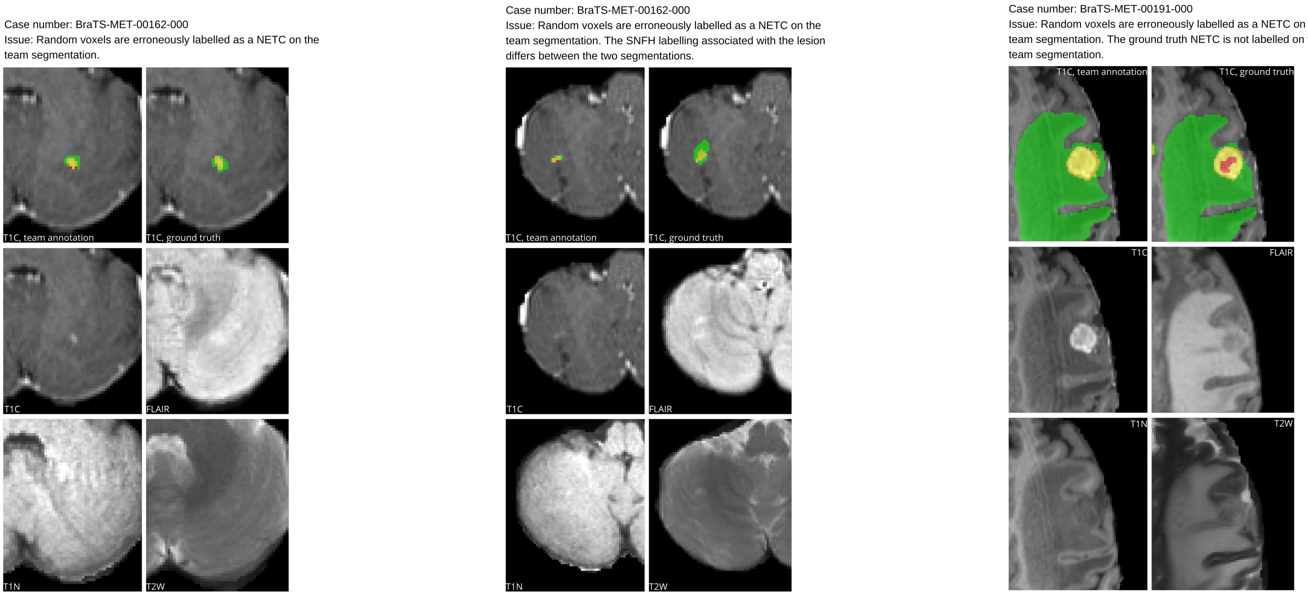

Figure 13: Supplementary: Examples of Random Voxels Predicted as Non-enhancing tumor core

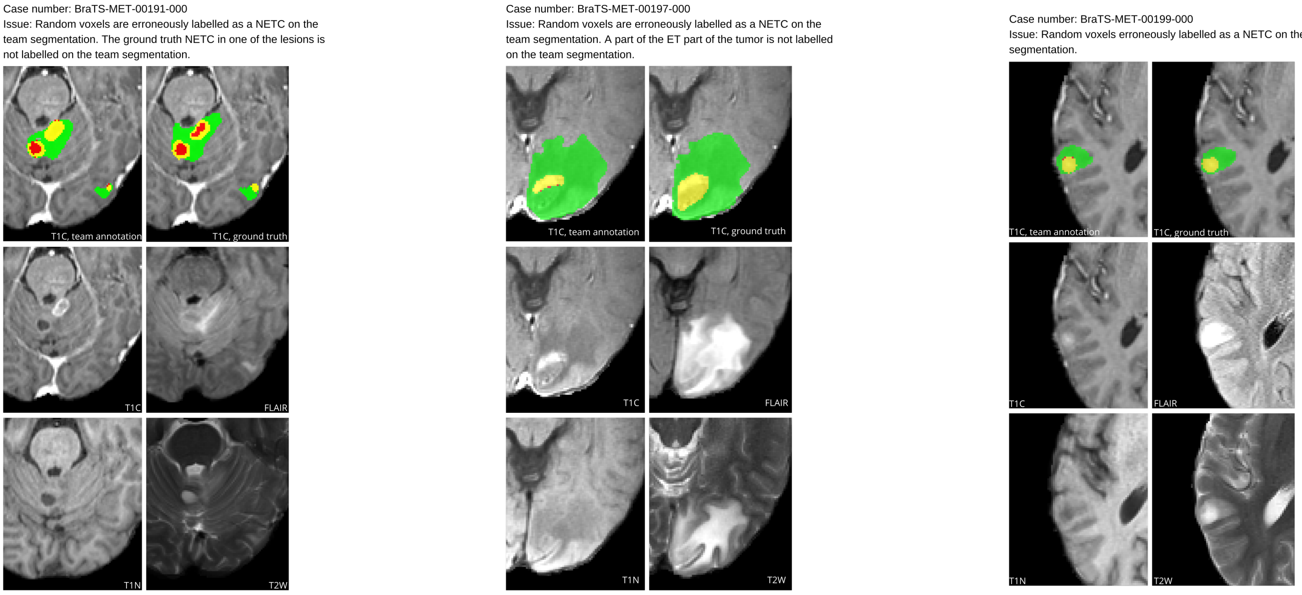

Figure 14: Supplementary: Examples of Random Voxels Predicted as Non-enhancing tumor core

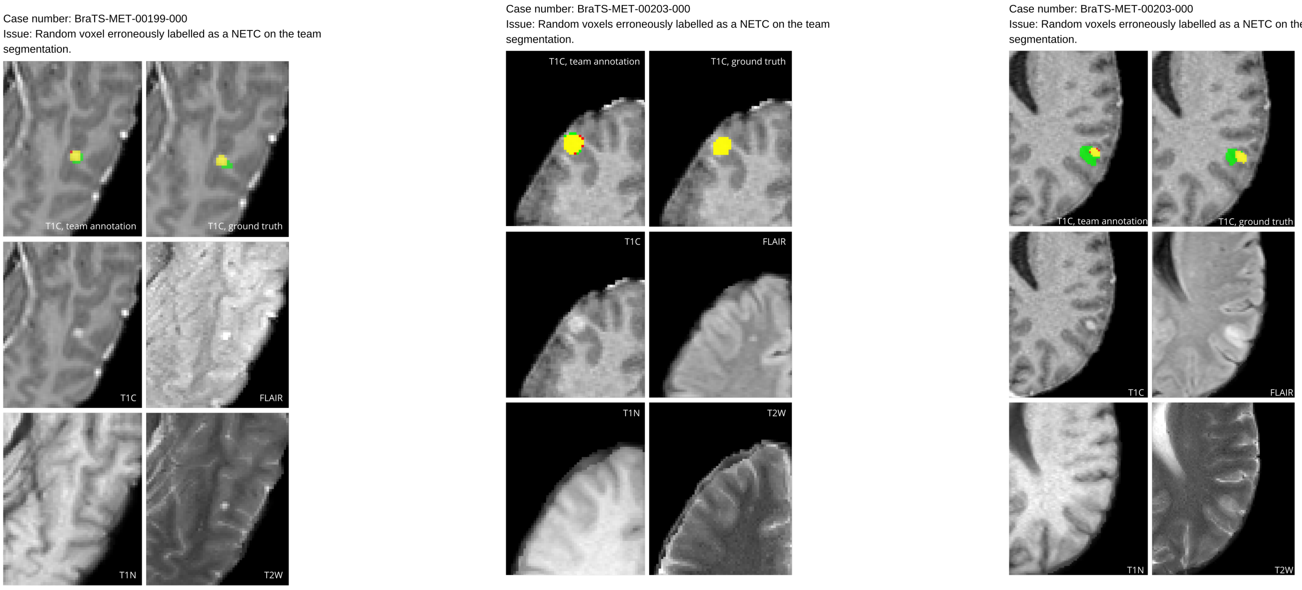

Figure 15: Supplementary: Examples of Random Voxels Predicted as Non-enhancing tumor core

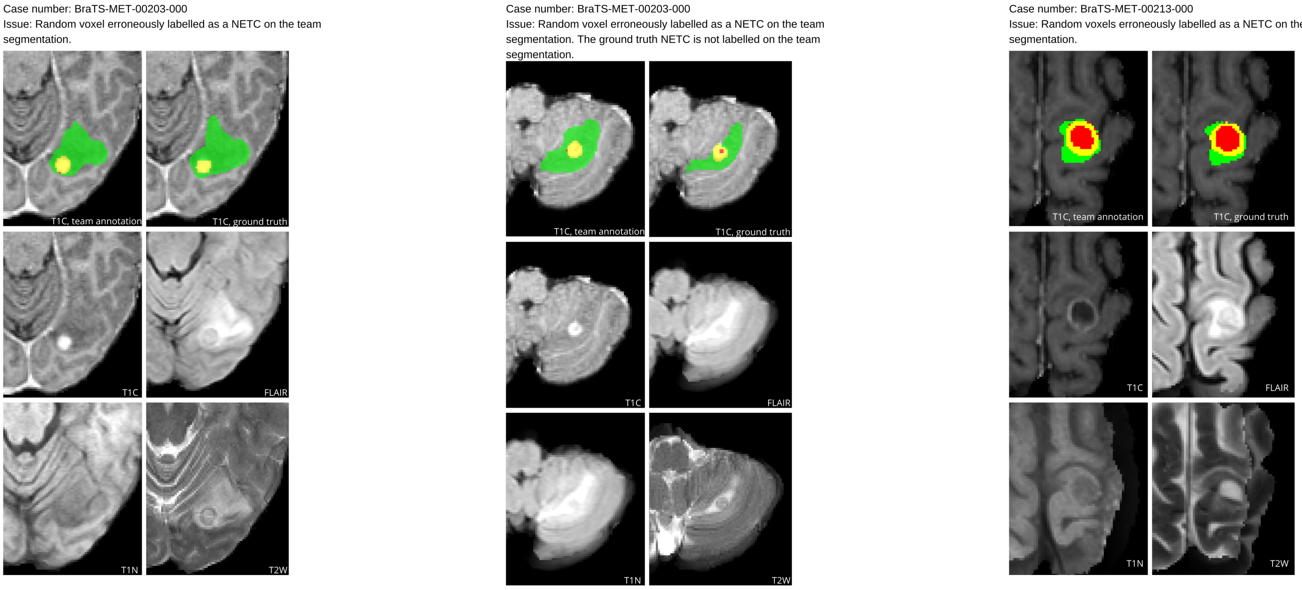

Figure 16: Supplementary: Examples of Random Voxels Predicted as Non-enhancing tumor core

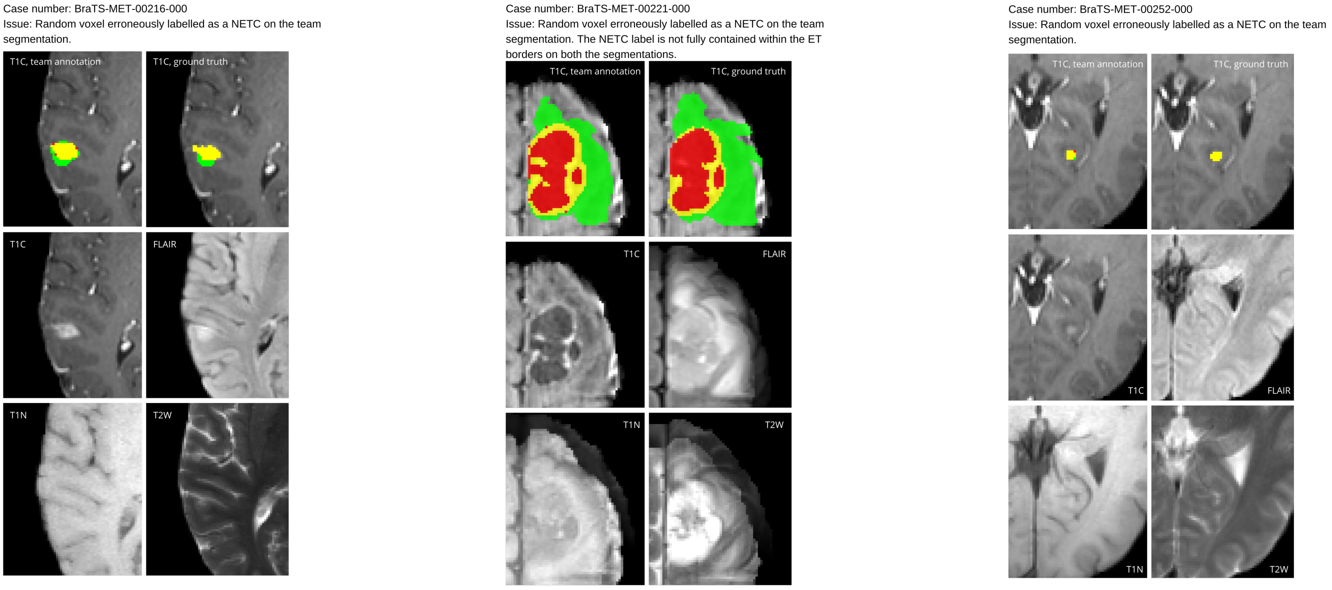

Figure 17: Supplementary: Examples of Random Voxels Predicted as Non-enhancing tumor core

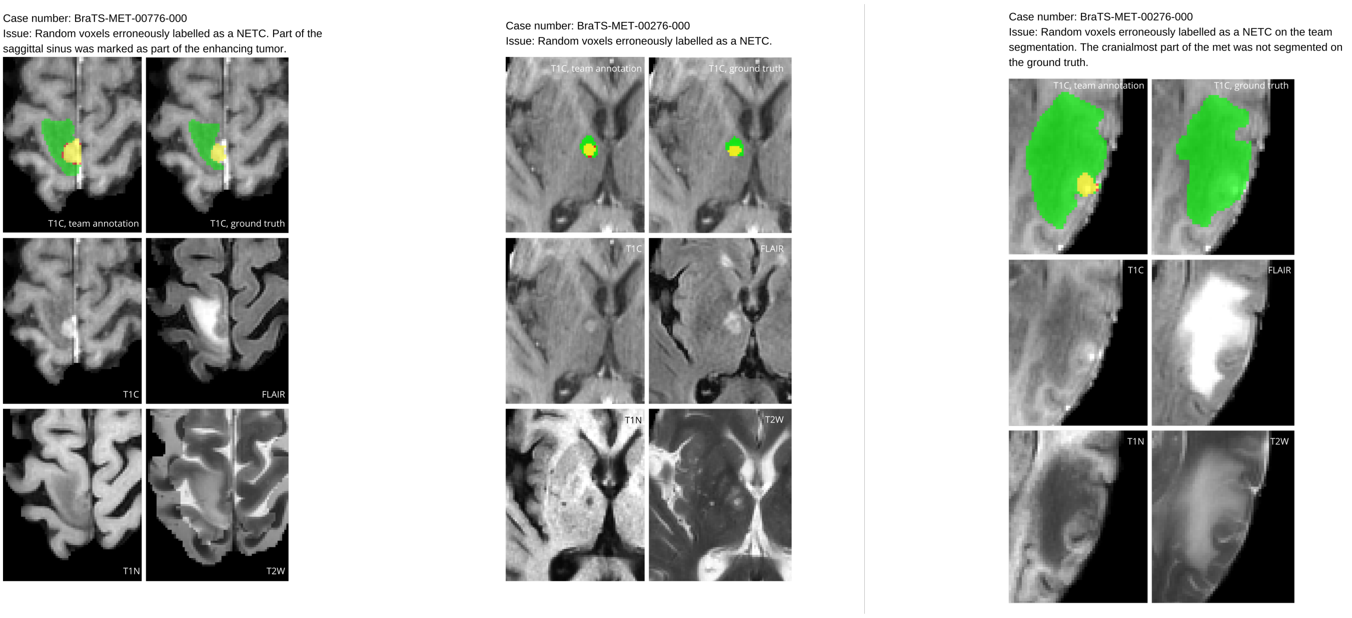

Figure 18: Supplementary: Examples of Random Voxels Predicted as Non-enhancing tumor core

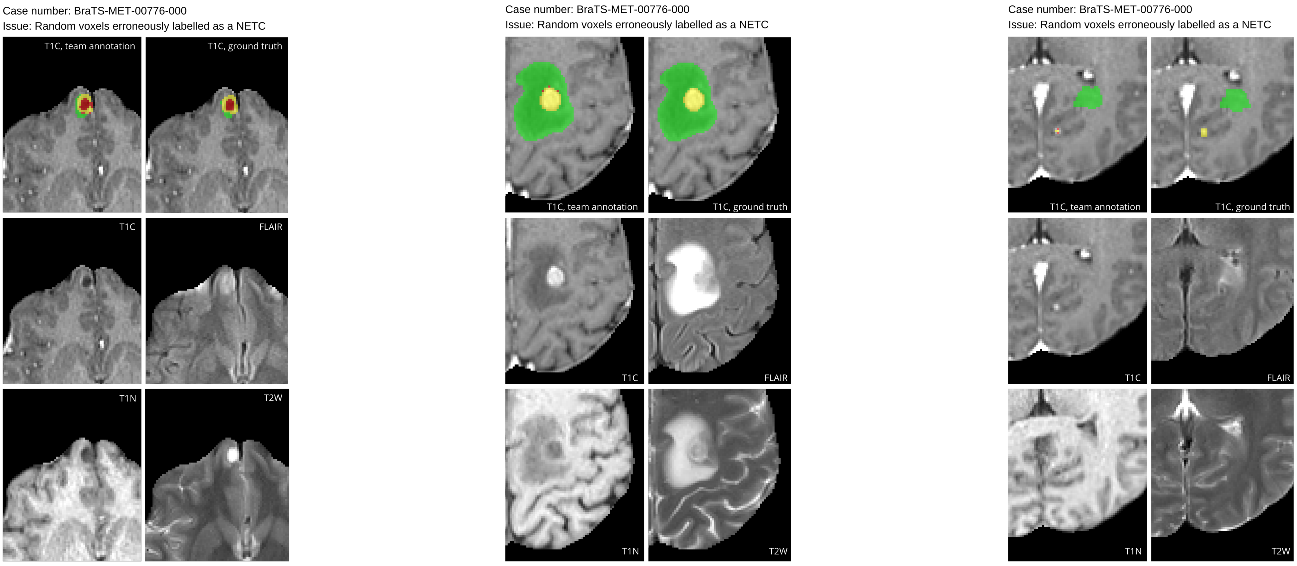

Figure 19: Supplementary: Examples of Random Voxels Predicted as Non-enhancing tumor core

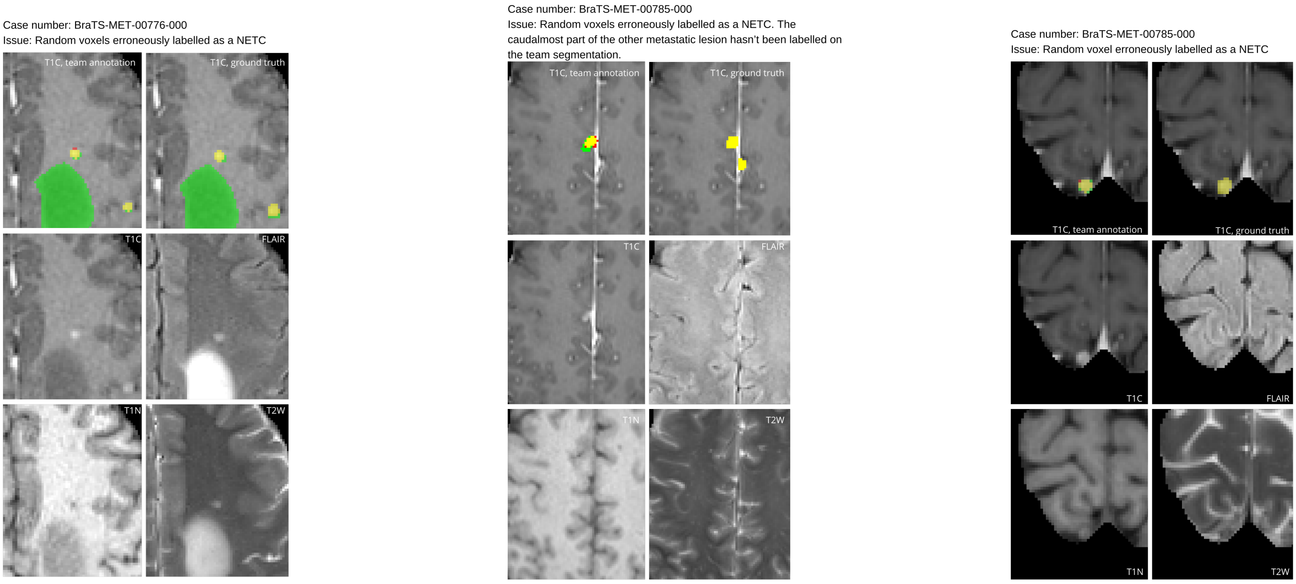

Figure 20: Supplementary: Examples of Random Voxels Predicted as Non-enhancing tumor core

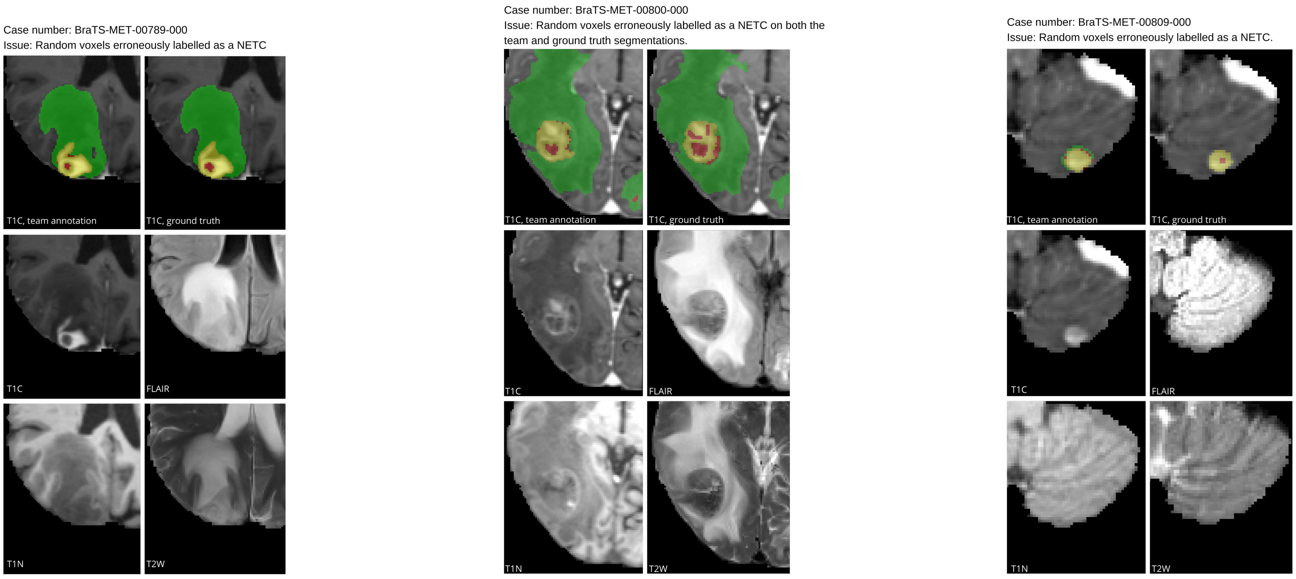

Figure 21: Supplementary: Examples of Random Voxels Predicted as Non-enhancing tumor core

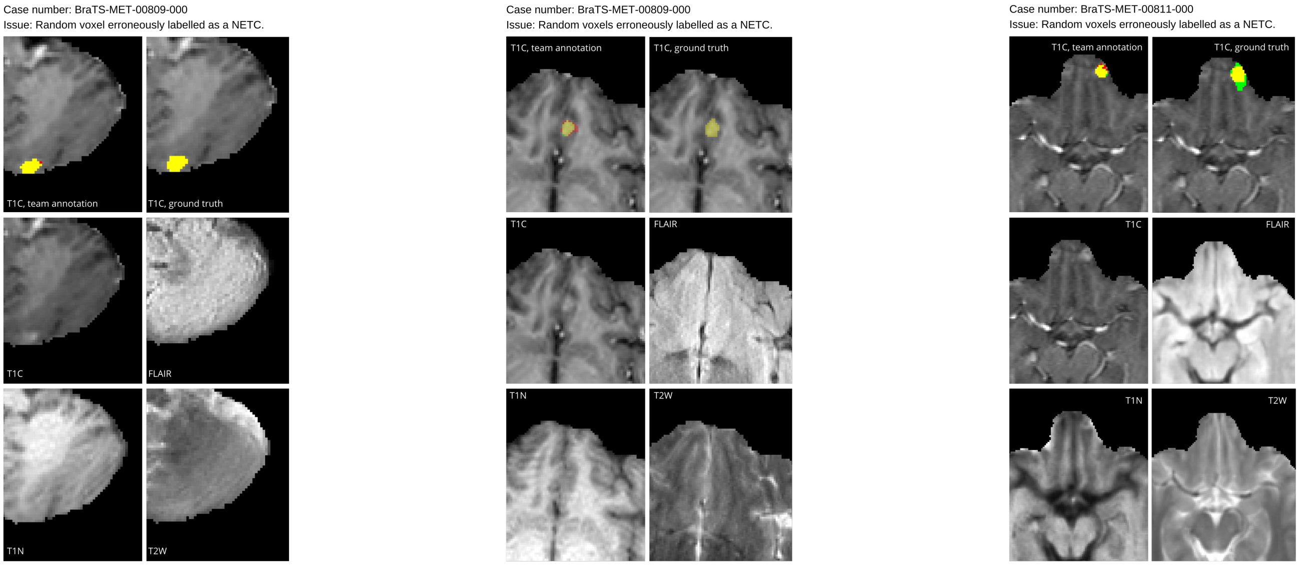

Figure 22: Supplementary: Examples of Random Voxels Predicted as Non-enhancing tumor core

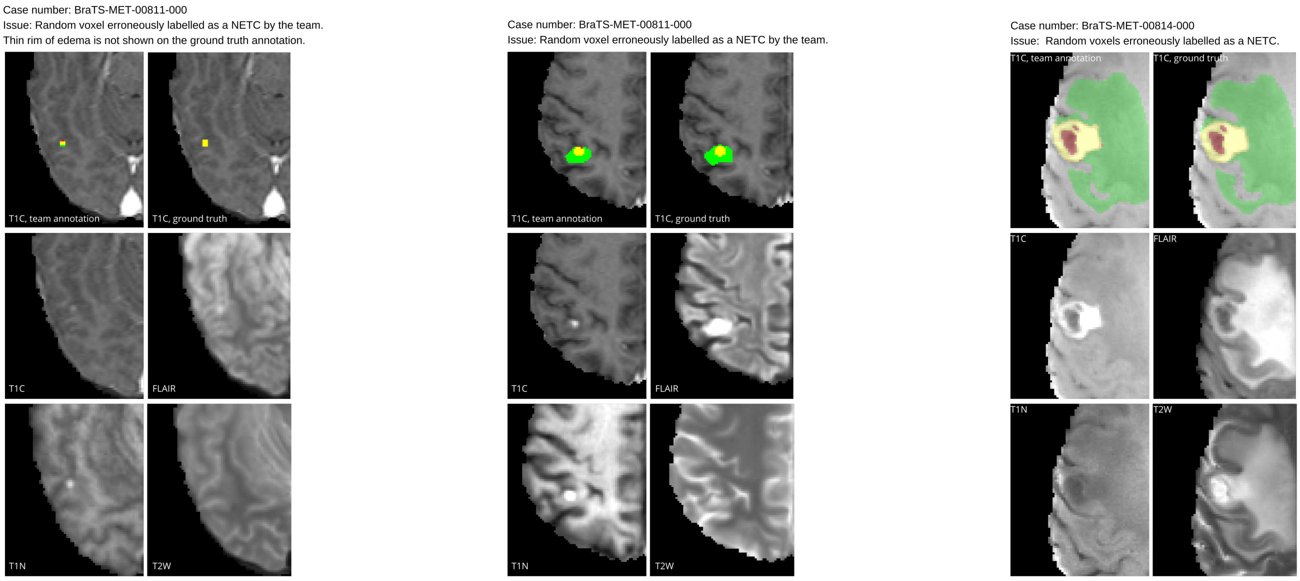

Figure 23: Supplementary: Examples of Random Voxels Predicted as Non-enhancing tumor core

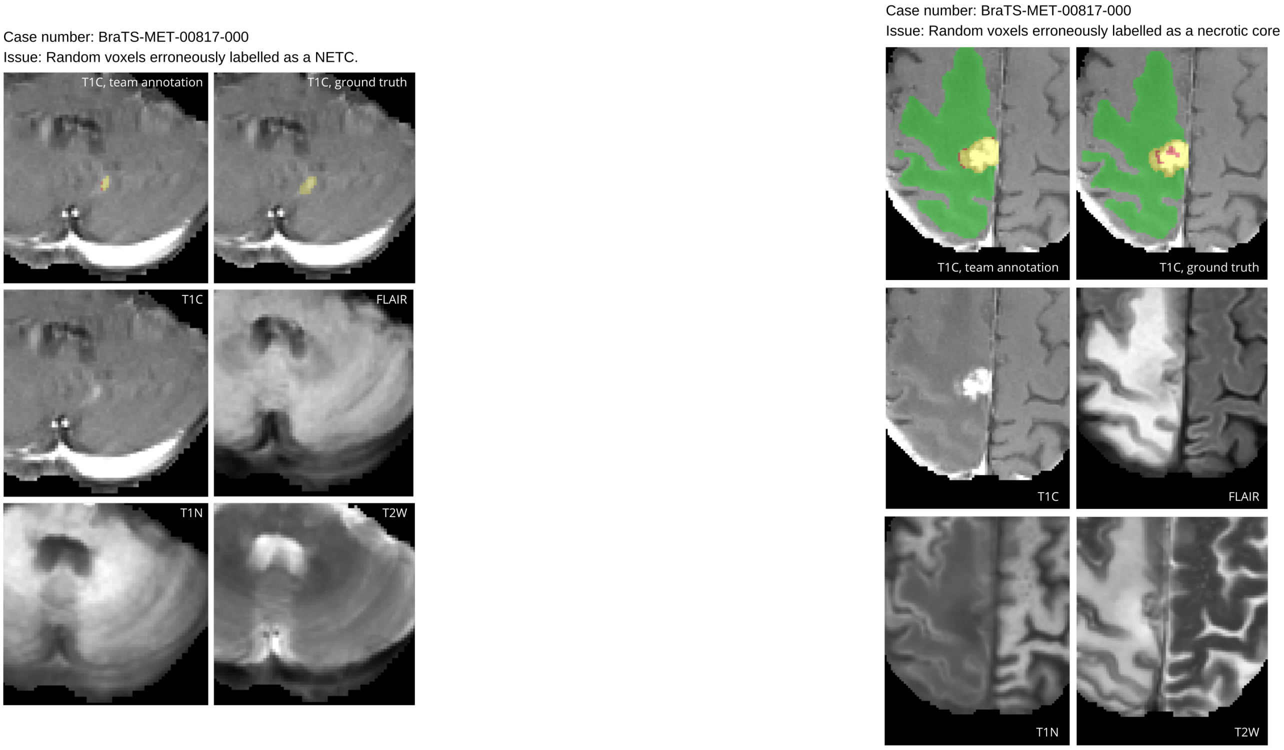

Figure 24: Supplementary: Examples of Random Voxels Predicted as Non-enhancing tumor core

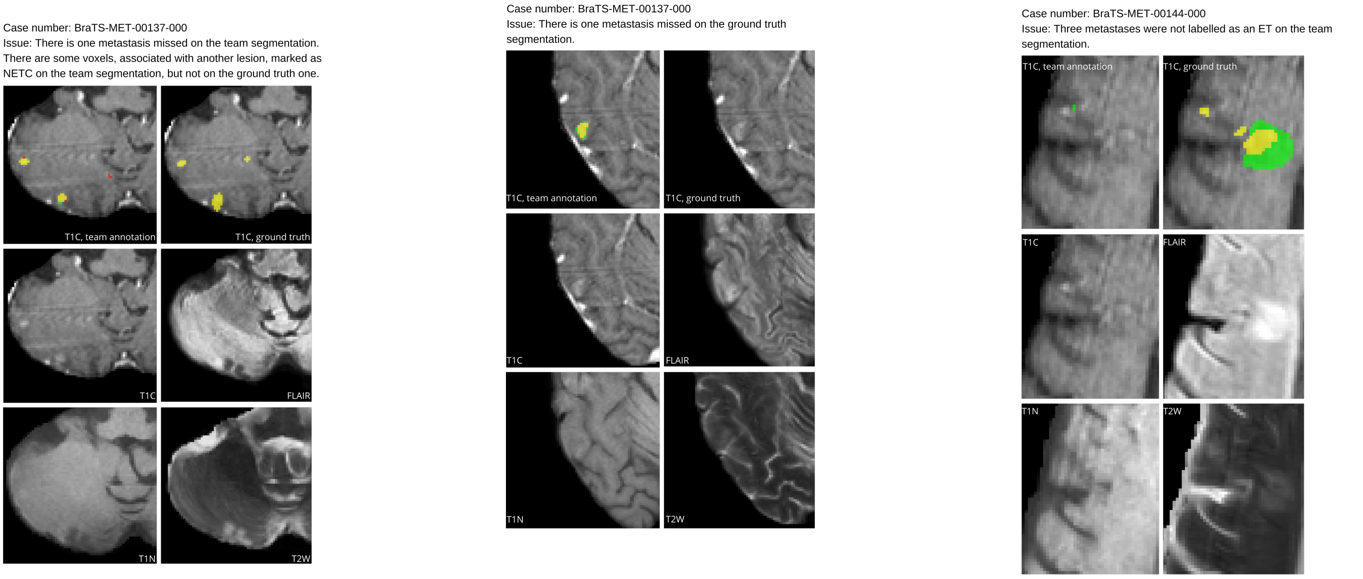

Figure 25: Supplementary: Pitfall Cases

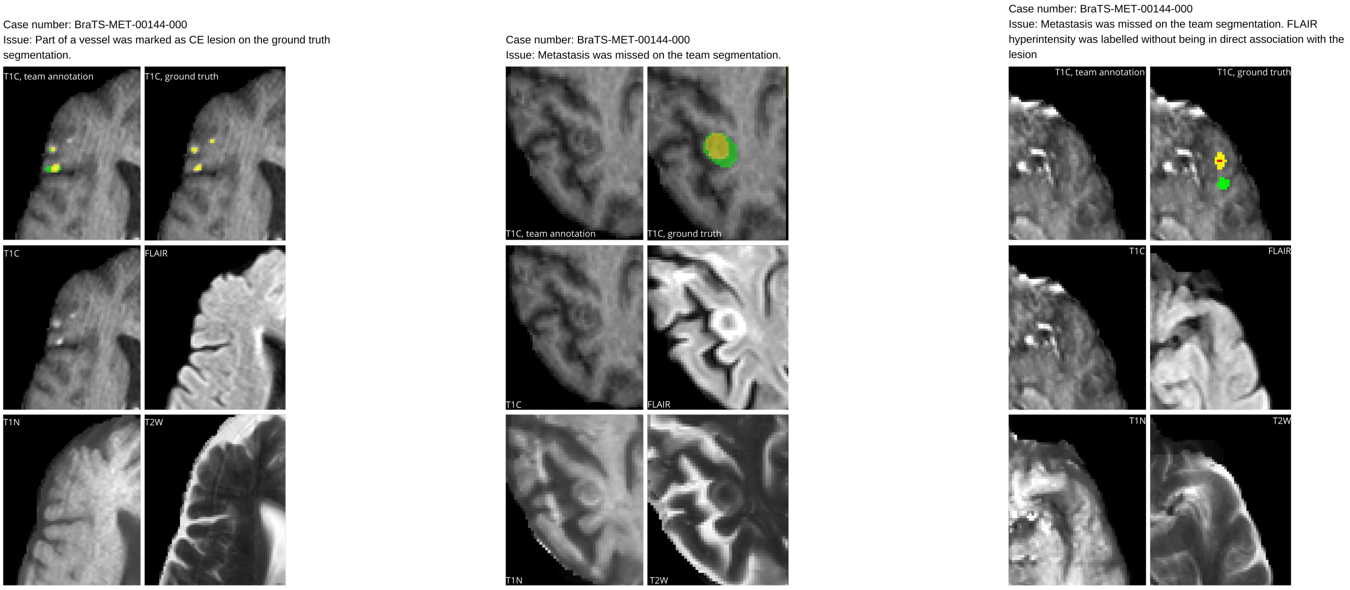

Figure 26: Supplementary: Pitfall Cases

Case number: BraTS-MET-00147-000
Issue: Metastasis was missed on the team segmentation.

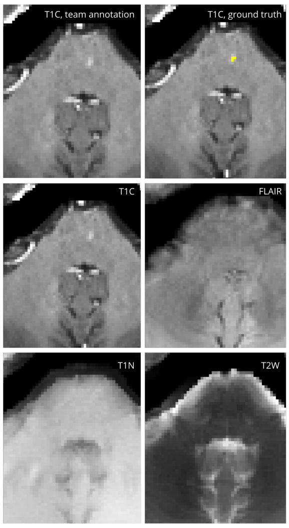

Case number: BraTS-MET-00148-000
Issue: Metastasis was missed on the team segmentation.

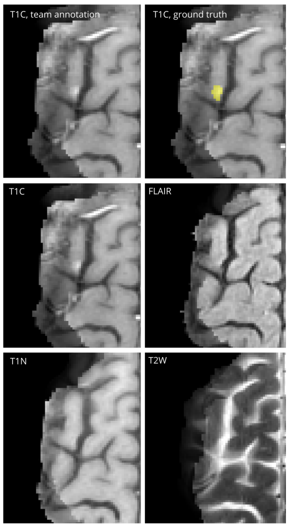

Case number: BraTS-MET-00148-000
Issue: Metastasis was missed on the team segmentation.

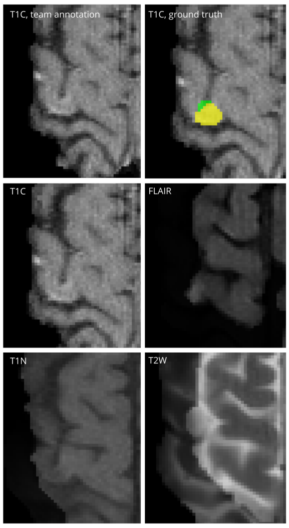

Figure 27: Supplementary: Pitfall Cases

Case number: BraTS-MET-00152-000
Issue: A vessel was erroneously marked as a small met on the team segmentation.

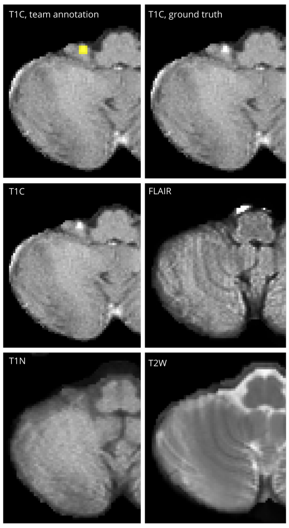

Case number: BraTS-MET-00162-000
Issue: Metastasis was missed on the ground truth segmentation.

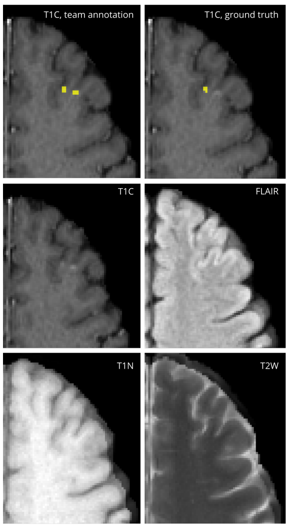

Case number: BraTS-MET-00162-000
Issue: Metastasis was missed on the ground truth and marked as NETC on the team segmentation.

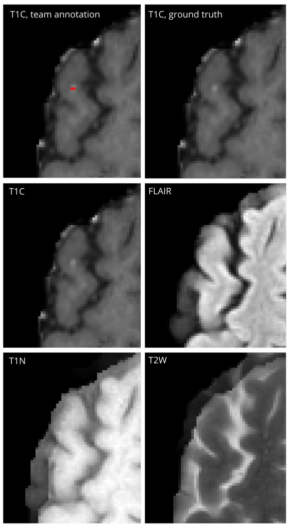

Figure 28: Supplementary: Pitfall Cases

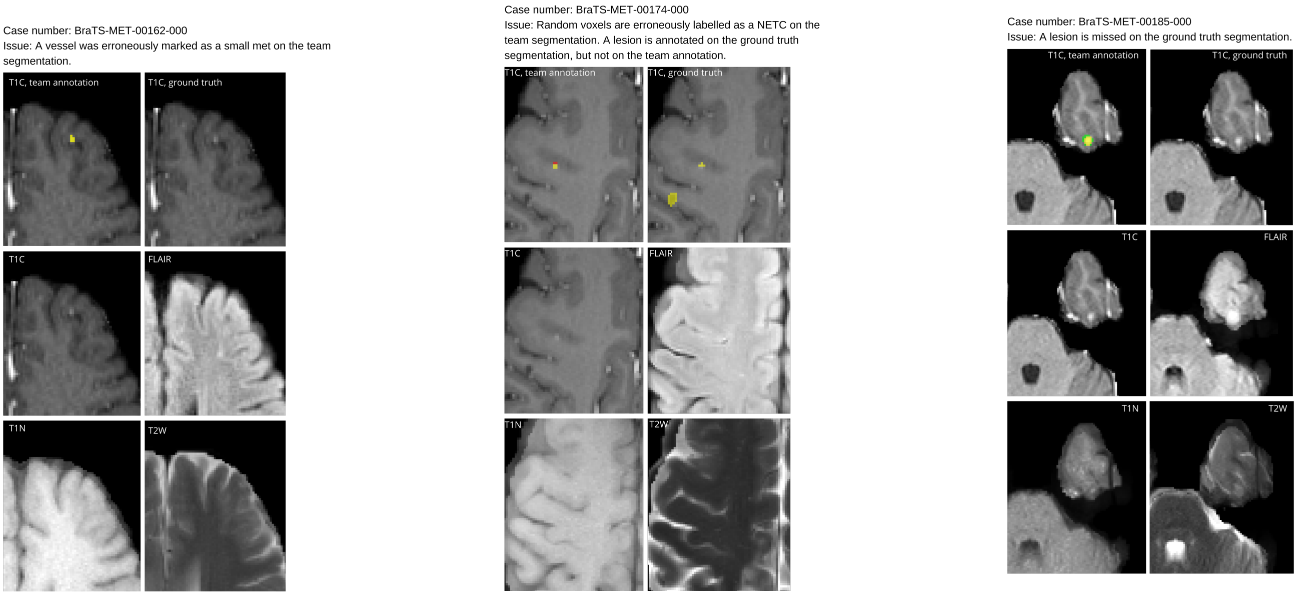

Figure 29: Supplementary: Pitfall Cases

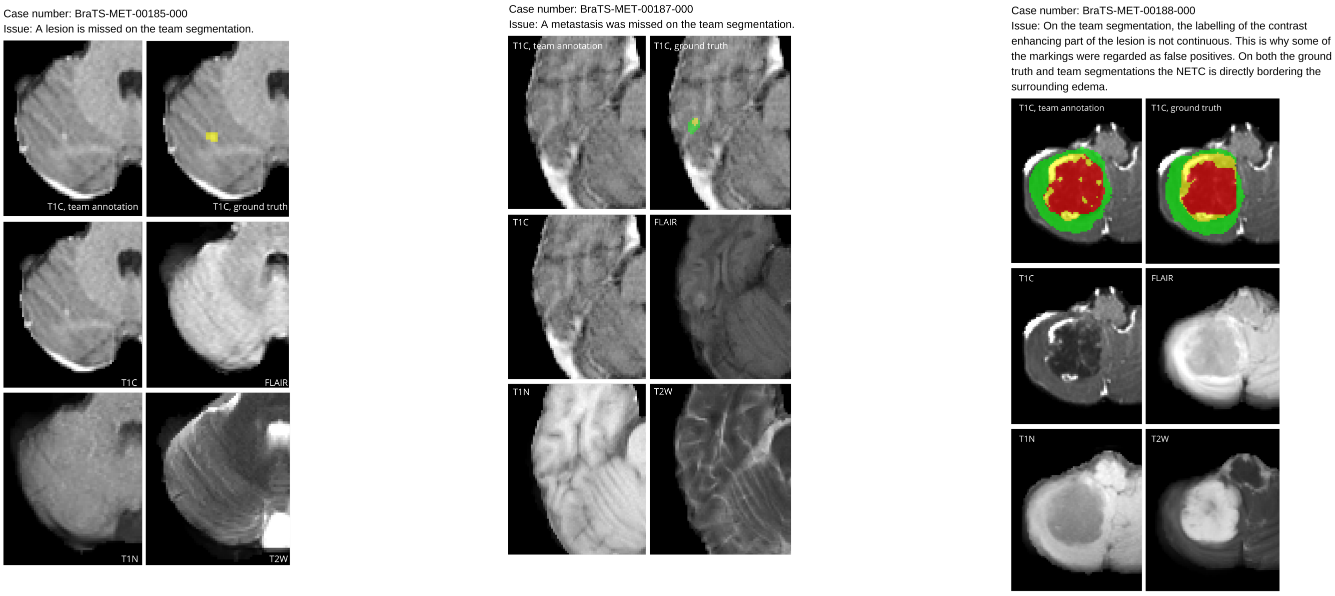

Figure 30: Supplementary: Pitfall Cases

Case number: BraTS-MET-00188-000
Issue: There was a metastatic lesion missed on the team segmentation.

Case number: BraTS-MET-00191-000
Issue: There was a metastatic lesion missed on the team segmentation.

Case number: BraTS-MET-00191-000
Issue: There was a metastatic lesion missed on the team segmentation.

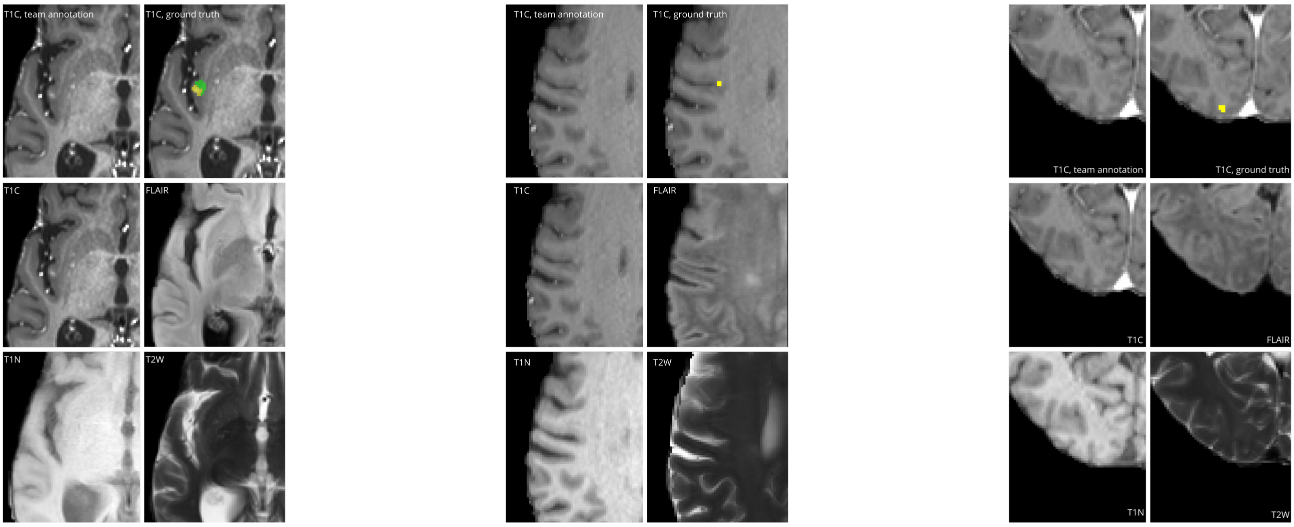

Figure 31: Supplementary: Pitfall Cases

Case number: BraTS-MET-00191-000
Issue: There was a metastatic lesion missed on the team segmentation.

Case number: BraTS-MET-00197-000
Issue: Metastasis was missed on the team segmentation.

Case number: BraTS-MET-00197-000
Issue: Metastasis was missed on the team segmentation.

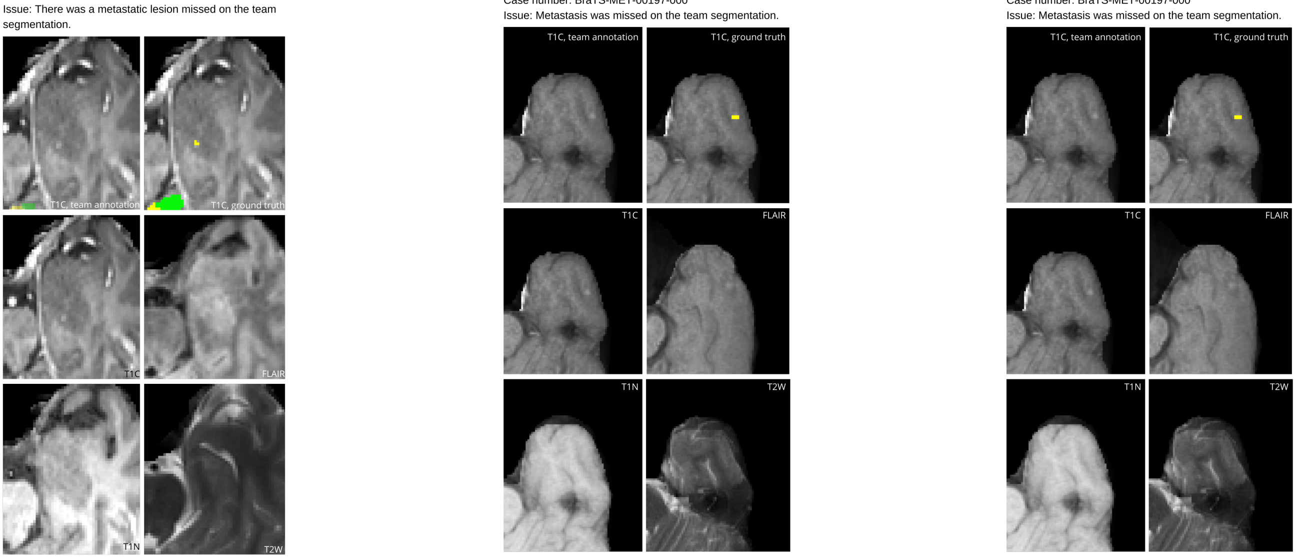

Figure 32: Supplementary: Pitfall Cases

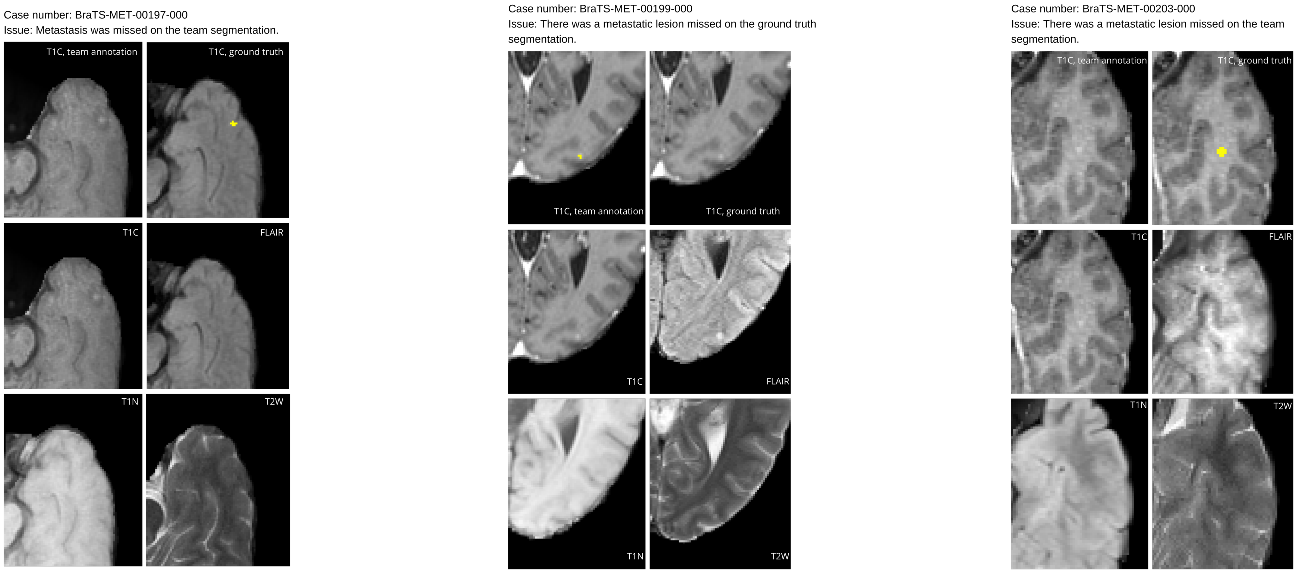

Figure 33: Supplementary: Pitfall Cases

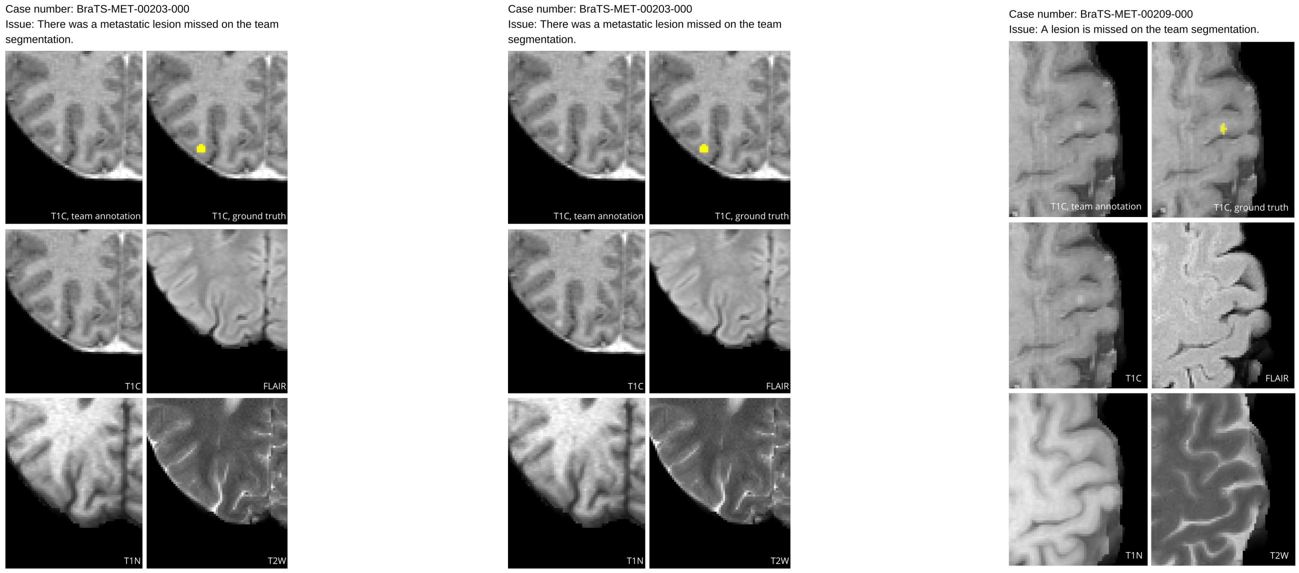

Figure 34: Supplementary: Pitfall Cases

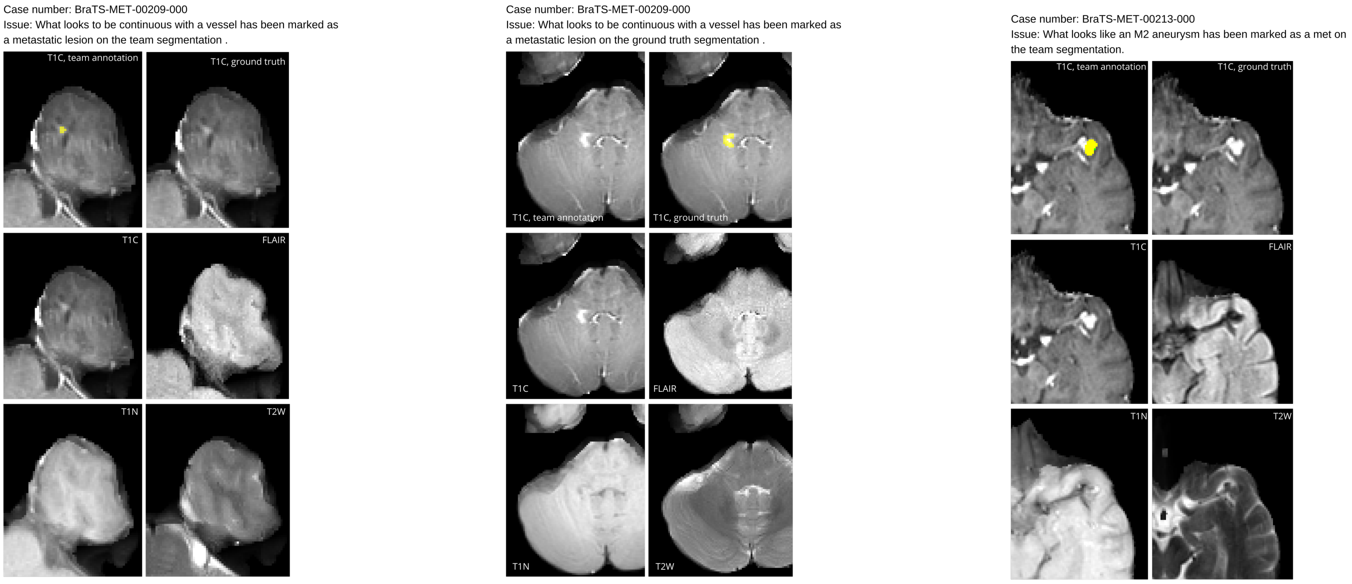

Figure 35: Supplementary: Pitfall Cases

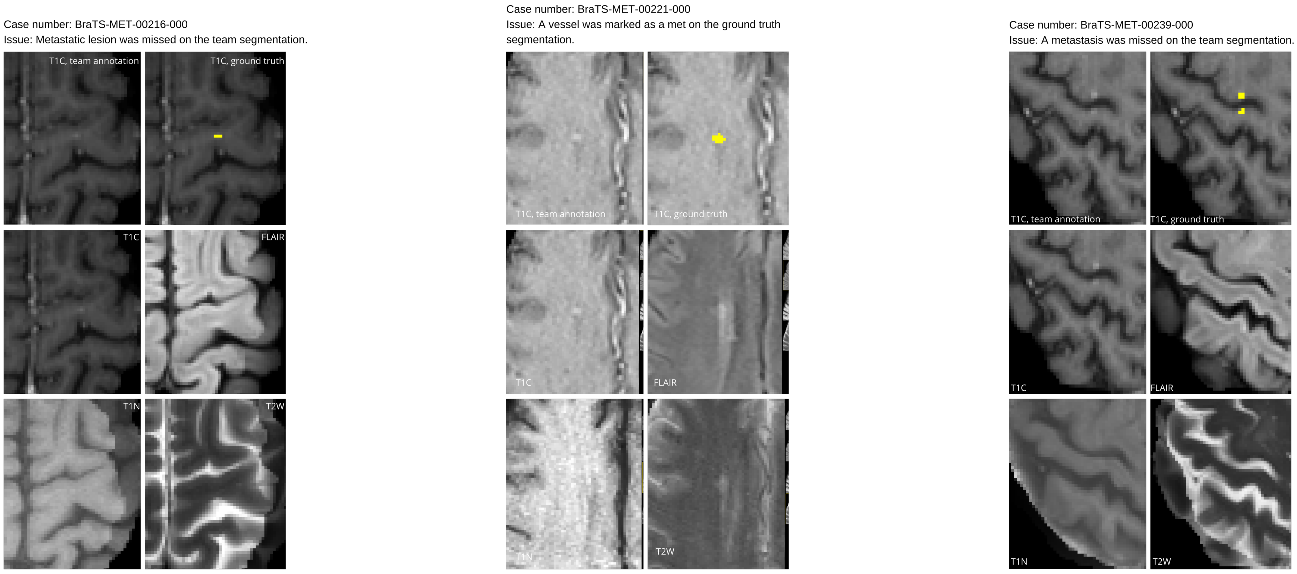

Figure 36: Supplementary: Pitfall Cases

Case number: BraTS-MET-00252-000
Issue: A metastasis was not labelled on the team segmentation.

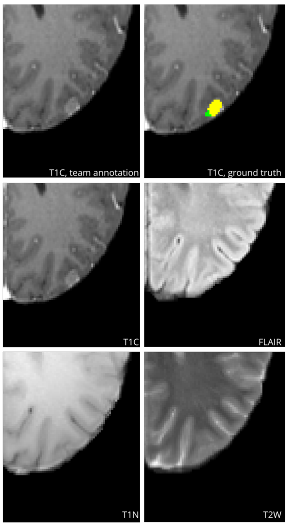

Case number: BraTS-MET-00276-000
Issue: A vessel was marked as a small met on the team segmentation.

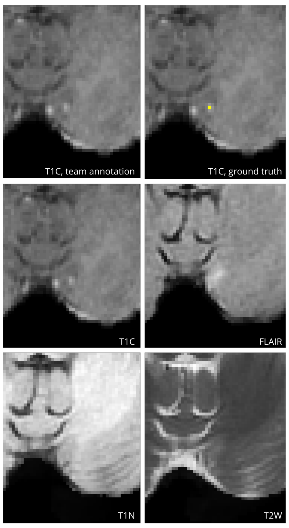

Case number: BraTS-MET-00776-000
Issue: A small metastasis was marked as SNFH

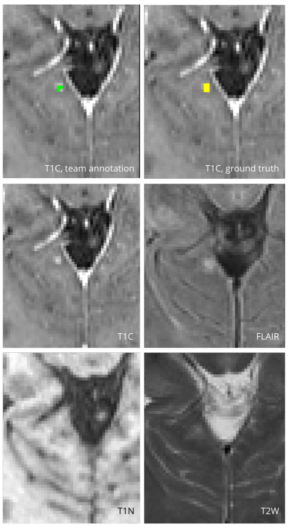

Figure 37: Supplementary: Pitfall Cases

Case number: BraTS-MET-00789-000
Issue: What looks like a part of a vessel has been marked as a small met on the ground truth segmentation.

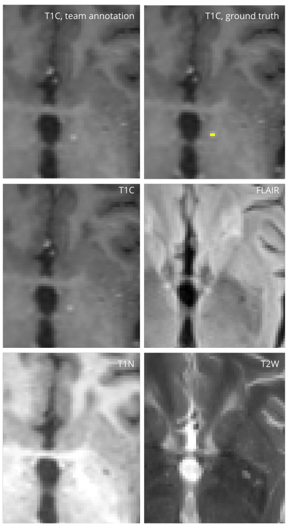

Case number: BraTS-MET-00800-000
Issue: Part of the signal from transverse-sigmoid sinus junction has been marked as ET on the ground truth segmentation.

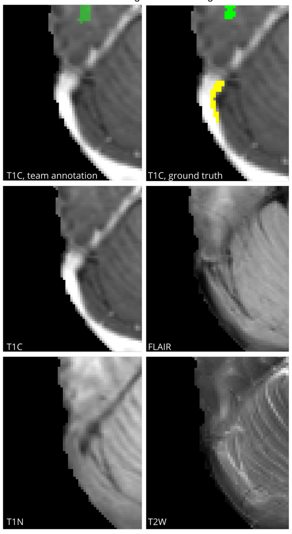

Case number: BraTS-MET-00809-000
Issues:
-One part of a metastatic lesion is labelled as edema on the team annotation (A) and another part of it is totally missed (B).
-On the ground truth image, some of the necrotic core is labelled in a way that suggests it is not contained within the ET part of the tumor (A). On another slice a couple of voxels within the lesion are not assigned neither to NETC, nor to ET (B).

A.

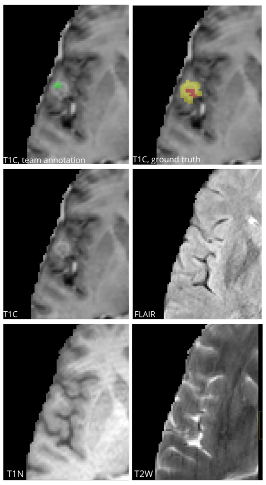

Figure 38: Supplementary: Pitfall Cases

B.

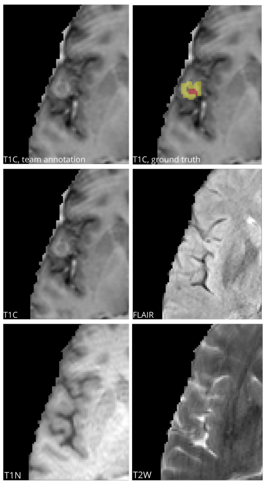

Case number: BraTS-MET-00809-000
Issue: Metastatic lesion erroneously marked as a NETC.

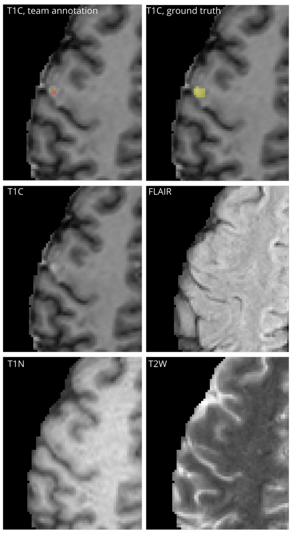

Case number: BraTS-MET-00809-000
Issues:
-Metastatic lesion protruding into the right lateral ventricle is not labelled on the team annotation image. However, on the ground truth annotation, the necrotic core is depicted as not being contained within the tumor.
-Metastatic lesion withing the superior cerebellar vermis hasn't been marked on the team annotation image.

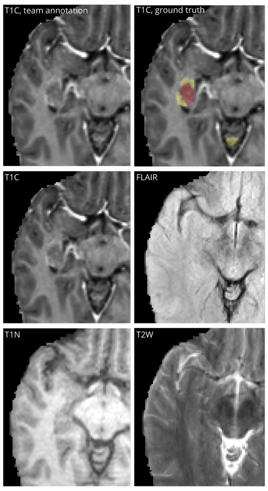

Figure 39: Supplementary: Pitfall Cases

Case number: BraTS-MET-00809-000
Issue: Missed small metastasis.

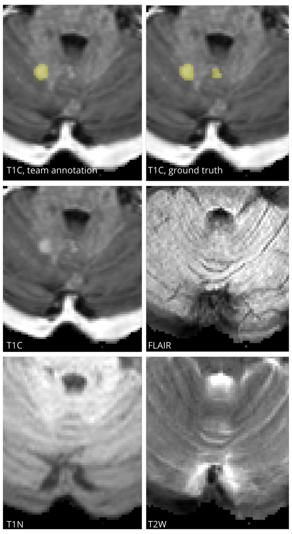

Case number: BraTS-MET-00809-000
Issue: A metastasis hasn't been labelled on the team segmentation.

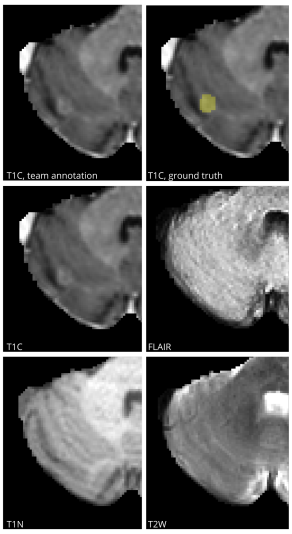

Case number: BraTS-MET-00809-000
Issue: The metastatic lesion is only partly segmented.

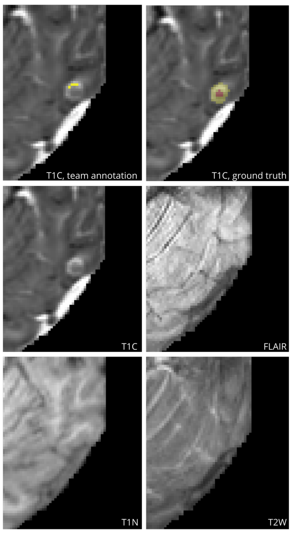

Figure 40: Supplementary: Pitfall Cases

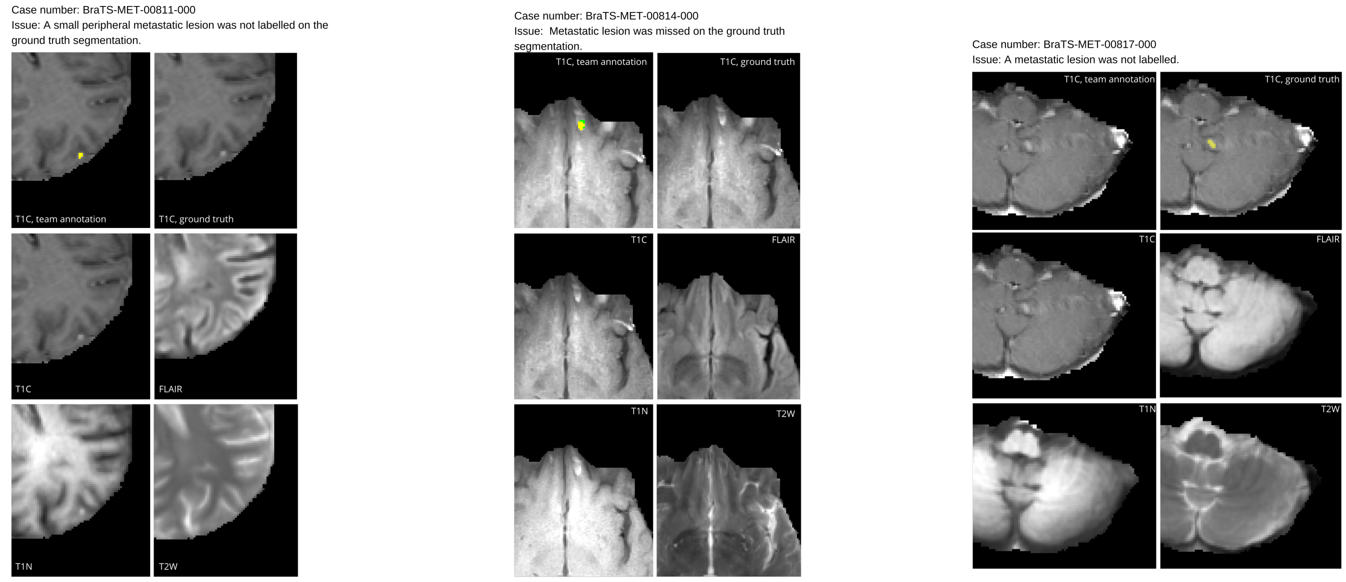

Figure 41: Supplementary: Pitfall Cases

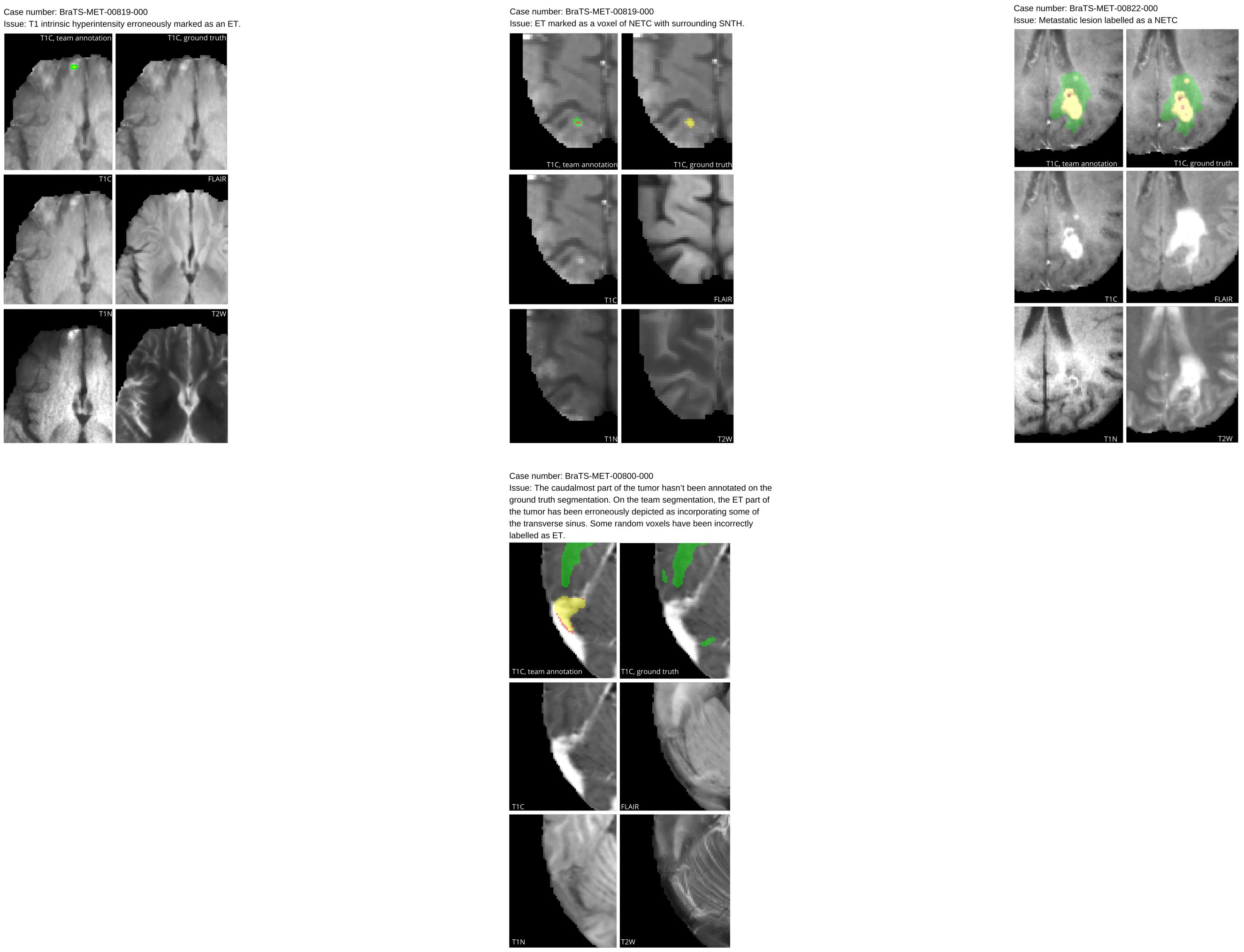

Figure 42: Supplementary: Pitfall Cases
